# Analysis of long noncoding RNA expression in hepatocellular carcinoma of different viral etiology

**DOI:** 10.1186/s12967-016-1085-4

**Published:** 2016-11-28

**Authors:** Quan Zhang, Kentaro Matsuura, David E. Kleiner, Fausto Zamboni, Harvey J. Alter, Patrizia Farci

**Affiliations:** 1 Laboratory of Infectious Diseases, Hepatic Pathogenesis Section, National Institute of Allergy and Infectious Diseases (NIAID), National Institutes of Health (NIH), Bethesda, MD 20892 USA; 2 Department of Transfusion Medicine, Warren G. Magnuson Clinical Center, NIH, Bethesda, MD 20892 USA; 3 Laboratory of Pathology, National Cancer Institute, National Institutes of Health, Bethesda, MD 20892 USA; 4 Liver Transplantation Center, Brotzu Hospital, Cagliari, Italy; 5 Department of Experimental Medicine and Infectious Diseases, Nanjing Drum Tower Hospital, Nanjing University Medical School, 321 Zhongshan Road, Nanjing, 210008 China

**Keywords:** Hepatocellular carcinoma, Long noncoding RNA, Hepatitis B virus, Hepatitis C virus, Hepatitis D virus

## Abstract

**Background:**

Dysregulation of long noncoding RNA (lncRNA) expression contributes to the pathogenesis of many human diseases, including liver diseases. Several lncRNAs have been reported to play a role in the development of hepatocellular carcinoma (HCC). However, most studies have analyzed lncRNAs only in hepatitis B virus (HBV)-related HCC or in a single group of HCC patients regardless of the viral etiology.

**Methods:**

To investigate whether lncRNAs are differentially expressed in HCC of different viral etiology, we profiled 101 disease-related lncRNAs, including 25 lncRNAs previously associated with HCC, in liver specimens obtained from well-characterized patients with HBV-, hepatitis C virus (HCV)-, or hepatitis D virus (HDV)-associated HCC.

**Results:**

We identified eight novel HCC-related lncRNAs that were significantly dysregulated in HCC tissues compared to their surrounding non-tumorous tissues. Some of these lncRNAs were significantly dysregulated predominantly in one specific hepatitis virus-related HCC, including PCAT-29 in HBV-related HCC, aHIF and PAR5 in HCV-related HCC, and Y3 in HDV-related HCC. Among the lncRNAs previously reported in HCC, we found that DBH-AS1, hDREH and hPVT1 were differentially expressed in HCC of different viral etiology.

**Conclusions:**

Our study suggests that HCC of different viral etiology is regulated by different lncRNAs. The identification of lncRNAs unique to specific hepatitis virus-related HCC may provide new tools for improving the diagnosis of HCC and open new avenues for disease-specific therapeutic interventions.

**Electronic supplementary material:**

The online version of this article (doi:10.1186/s12967-016-1085-4) contains supplementary material, which is available to authorized users.

## Background

Recent findings from genome tiling arrays and RNA sequencing have revealed the existence of a large number of RNAs that lack protein-coding capacity, which can be divided into two groups, namely, small non-coding RNAs (sncRNA) (<200 bp) and long non-coding RNAs (lncRNAs) (>200 bp) [1, 2]. Although lncRNAs have long been viewed as transcriptional “noise”, increasing evidence shows that they function in many cellular processes and may play a role in the pathogenesis of cancer and other diseases [3, 4]. LncRNAs are involved in both transcriptional and post-transcriptional regulation, and can contribute structural/scaffolding functions [1].

Hepatocellular carcinoma (HCC) is the fifth most common cancer worldwide and the second cause of cancer-related death [5]. Chronic infections with hepatitis B virus (HBV), hepatitis C virus (HCV) and hepatitis D virus (HDV) account for over 80% of HCC cases [6]. It has been estimated that about a half billion people are chronically infected with hepatitis viruses worldwide (250 million with HBV, 170 million with HCV, and 15 million with HDV) [7–9]. Studies on the natural history of chronic viral hepatitis have shown that over a period of 10–40 years about 30% of patients will develop cirrhosis and its long-term consequences, liver decompensation and/or HCC [10], leading to liver-related death or liver transplantation. Although an etiological link between hepatitis viruses and HCC has been well established, the molecular mechanisms whereby hepatitis viruses induce liver cancer remain to be elucidated.

Previous studies on the molecular pathogenesis of HCC were mainly focused on investigating the role of protein-coding genes. However, there is increasing interest in the study of non-coding RNAs, including sncRNAs and lncRNAs [11]. Although extensive studies have been performed on sncRNAs, in particular microRNAs (miRNAs), our understanding of the lncRNA functions in HCC is still limited. Several lncRNAs have been shown to be involved in the development of HCC, providing new insights into pathogenesis and highlighting lncRNAs as potential diagnostic, prognostic and therapeutic factors in this cancer [12]. However, a major limitation of previous studies is that they either analyzed lncRNAs selectively in HBV-related HCC or considered HCC as a single group regardless of the hepatitis virus involved. In this study, we analyzed the expression of lncRNAs in HBV-, HCV-, and HDV-related HCC patient samples, with the aim of investigating the differential role of lncRNAs in relation to the different viral etiology of HCC.

## Methods

### Patients

We studied a total of 63 patients. Twenty-five patients had viral-associated HCC. The etiology was infection with HBV in 11 patients, HCV in 10 and HDV in 4. Twenty had non-HCC cirrhosis; the etiology was infection with HBV in 3, HCV in 10 and HDV in 7. The control group included 10 liver donors and 8 subjects who underwent liver resection for hepatic hemangioma. All patients were negative for human immunodeficiency virus type-1. For each liver biopsy specimen, the stage of fibrosis and the activity grade were evaluated according to the Ishak scoring system [13]. The grade of tumor differentiation was established according to the Edmondson and Steiner grading system [14]. Patients were followed at the Liver Transplantation Center of the Brotzu Hospital in Cagliari, Italy. All patients provided written informed consent, and the protocol was approved by the ethical Committee of the Hospital Brotzu, Cagliari, Italy. The study was also approved by the Office of Human Subjects Research of the National Institutes of Health, Bethesda, MD, on the condition that all samples were de-identified.

### Liver specimens for the expression of long-noncoding RNAs

**Fig. 1:**
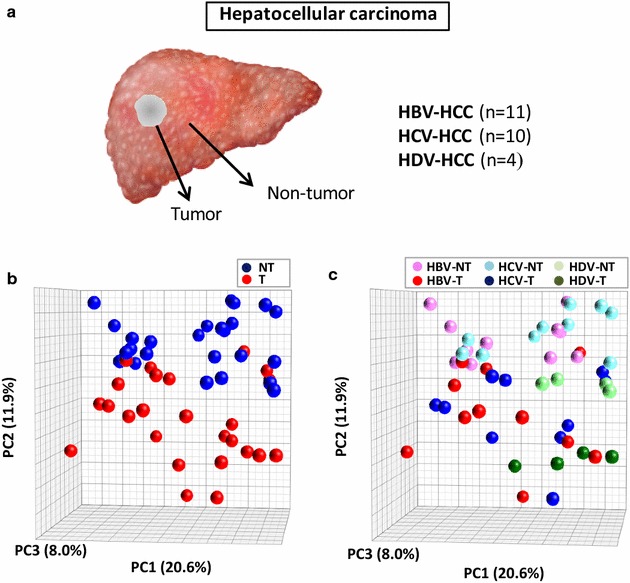
Study design and principal component analysis showing the relationship among the tissue groups in patients with HCC of different viral etiology. The analysis was based on the results of the normalized expression levels of 68 long non-coding RNAs (lncRNA) detected in tumor tissues and their surrounding non-tumorous tissues using disease-related human LncRNA Profiler. **a** Study design illustrating the different groups of patients included in the profiling of the expression of disease-related lncRNAs. HCC, hepatocellular carcinoma; HBV, hepatitis B virus; HCV, hepatitis C virus; HDV, hepatitis D virus. **b** Principal component analysis of tumor (*T*) and surrounding non-tumor tissue (*NT*). **c** Principal component analysis of tumor (*T*) and surrounding non-tumor tissue (*NT*) of 6 groups of patients according to the viral etiology. Each principal component contribution rate is shown in each axis. *PCA* Principal component analysis; *PC* principal component

**Fig. 2:**
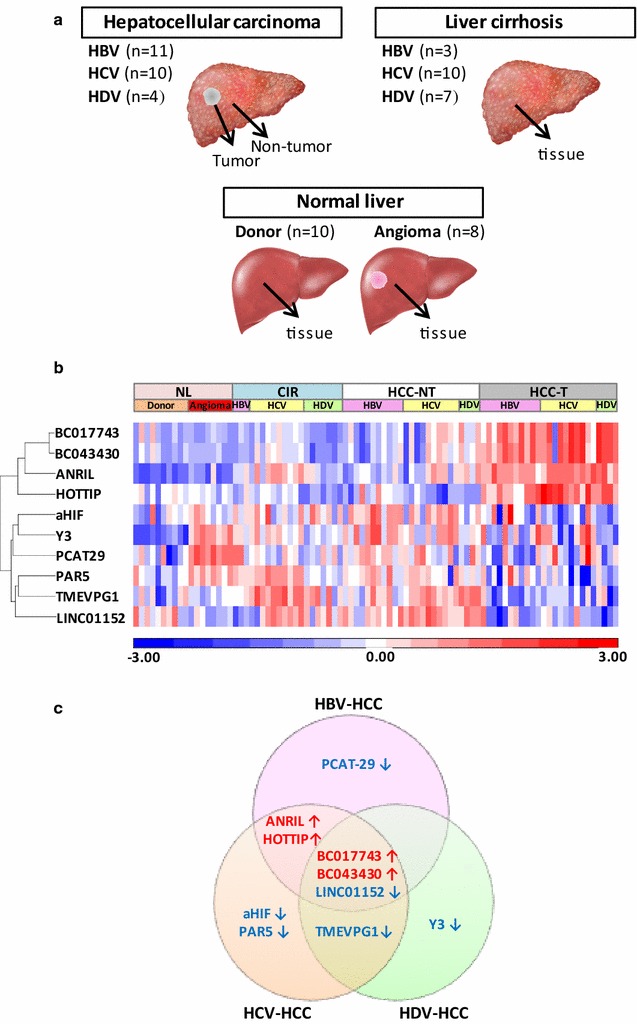
Differentially expressed lncRNAs in HBV-, HCV-, and HDV-related HCC, non-HCC cirrhosis and normal livers. **a** Study design illustrating the different groups of patients. **b** Hierarchical clustering based on the 10 lncRNAs differentially expressed in HBV-, HCV-, and/or HDV-related HCC. LncRNAs that are up-regulated are shown in shades of * red*; those down-regulated are shown in* shades of blue*. The intensity of the* color in each cell* reflects the level of the corresponding lncRNA in the corresponding patient expressed as normalized log_2_ ratios. *HCC-T*, denotes hepatocellular carcinoma; *HCC-NT*, denotes surrounding non-tumorous tissue; *CIR*, denotes cirrhosis without *HCC*; NL, denotes normal liver from liver donors or liver resection for angioma. **c** Summary of dysregulatd lncRNAs in different hepatitis viruses-associated HCC tissues compared to their surrounding non-tumorous tissues. Names of up-regulated lncRNAs in HCC are shown in* red* and those down-regulated are shown in *blue*

Among the 25 individuals with viral-associated HCC who underwent liver transplantation, two liver specimens were obtained from each patient, including one from the tumor and one from the surrounding non-tumorous tissue, while individual specimens were obtained from 20 patients with non-HCC cirrhosis of different viral etiology who had never developed HCC and underwent liver transplantation for end-stage liver disease, and from 18 patients of the control group. Thus, a total of 88 liver specimens were analyzed for the expression of lncRNAs (Figs. 1a, 2a). Each individual liver biopsy was divided into two pieces: one was snap-frozen and stored at −80°C for molecular studies and the other was formalin-fixed and paraffin-embedded for pathological examination. A critical point of our study is that confounding factors, such as the presence of a mixed population of tumor and non-tumor hepatocytes were completely ruled out, as all liver specimens were evaluated by an expert liver hepatopathologist and liver specimens containing a mixed cellular population were excluded from this study.

### RNA extraction

Total RNA was extracted from frozen liver specimens using the miRNeasy Mini Kit (Qiagen, Hilden, Germany). The concentration of total RNA was measured using a Nano Drop method (Nano Drop Technologies, Wilmington, DE). The quality and integrity of RNA were assessed with the RNA 6000 Nano Assay on the Agilent 2100 Bioanalyzer (Agilent Technologies, Santa Clara, CA).

### LncRNAs profiling

Extracted RNA was converted to cDNA using the ProtoScript® First Strand cDNA Synthesis Kit (New England Biolabs, Ipswich, MA). Disease-Related Human LncRNA Profiler (System Biosciences, Palo Alto, CA), which is an array based on quantitative real-time PCR (qRT-PCR), was used to profile lncRNAs in the samples according to the manufacturer’s instructions. Eighty-three lncRNAs profiled were as follows: 21A, AAA1, aHIF, AK023948, ANRIL, anti-NOS2A, BACE1AS, BC017743, BC043430, BC200, BCMS, BIC, CCND1ANCR, DD3, DGCR5, DISC2, DLG2AS, EGO, GAS5, GOMAFU, H19, H19-AS, HAR1A, HAR1B, HOTAIR, HOTAIRM1, HOTTIP, HOXA1ASAA489505, HOXA3ASBI823151, HOXA3ASBE873349, HOXA6ASAK092154, HOXA11AS, HULC, IPW, IGF2AS, KRASP1, L1PA16, LIT, LOC285194, LUST, LINC01152, LincRNAVLDLR, LincRNASFMBT2, MALAT1, MEG3, MER11C, NEAT1, NCRMS, NDM29, PANDA, PAR5, PCAT-1, PCAT-14, PCAT-29, PCAT-32, PCAT-43, PCGEM1, PR-AT2, PRINS, PSF inhibiting RNA, PTENP1, RMRP, ROR, SAF, SCA8, Sox2OT, SRA, ST7OT1, ST7OT2, ST7OT3, ST7OT4, Telomerase RNA, TMEVPG1, TU_0017629, TUG1, UCA1, WT1-AS, Y1, Y3, Y4, Y5, ZEB2NAT, 7SK. Eleven genes (7SL scRNA, 5.8S rRNA, U87 scaRNA, U6 smRNA, ACTB, B2 M, PGK1, GAPDH, HPRT1, RPL1A, RPL13A) were used as endogenous controls. We also analyzed an additional 18 HCC-related lncRNAs by qRT-PCR using the same primers as previously reported [15–32] and listed in Additional file 1: Table S1, including: AFAP1-AS1, CCAT1, DANCR, DBH-AS1, hDREH, GAS5, HEIH, LET, Linc00152, LincTCF7, MVIH, PCNA-AS1, hPVT1, uc.338, UCA1, UFC1, ZEB-AS1 and ZFAS1.

### Quantitative real-time PCR

Quantitative real-time PCR (qRT-PCR) was performed using the iQ SYBR Green Supermix (Bio-Rad, Hercules, CA) according to the manufacturer’s instructions. GAPDH was used as an endogenous control. LncRNAs expression levels were normalized by calculating the lncRNAs/GAPDH expression ratio (2^−ΔCt^). The relative expression of lncRNAs was calculated as the ratio between lncRNAs expression levels (2^−ΔCt^) in each liver specimen and the geometric mean of all normal livers. The primer sequences were listed in Additional file 1: Table S1. Amplification of qRT-PCR was carried out as follows: 95 °C for 3 min, followed by 40 cycles at 95 °C for 15 s and 60 °C for 60 s.

### Principal component analysis and hierarchical clustering

The expression levels of lncRNAs by the profiling or qRT-PCR were converted to log_2_ scale and imported into Partek Genomics Suite (Partek Inc., St. Louis, MO). The relationships between the exclusive 68 lncRNAs and HCC were visualized by principal component analysis on the samples and hierarchical clustering on the 10 dysregulated genes, in which dissimilarity was measured by the Euclidean distance and the average linkage method was used for clustering.

### Statistical analysis

The differences between groups for the matched samples were estimated by Student’s paired *t* test (two-tailed). The one-way analysis of variance (ANOVA) was used to determine the differences among the three groups. *P* value <0.05 was considered significant in all tests.

## Results

### Patients

The demographic, biochemical, virological, and histopathological features of the patients with viral-associated HCC or non-HCC cirrhosis included in the study are shown in Additional file 2: Table S2. There were no significant differences in the distribution of gender and age; in all groups, the vast majority were male. The levels of serum aminotransferases and γ-glutamyltransferase showed the lowest values in patients with HBV-associated HCC, whereas the highest values were observed in HCV-associated HCC; HDV showed intermediate values. The levels of α-fetoprotein were instead higher in HBV-associated HCC compared to HCV or HDV-associated HCC (Additional file 2: Table S2). The grade of tumor differentiation was G2 in 13 patients, G3 in 11, and G4 in 1; HCC was surrounded by a cirrhotic liver in all but three patients with HCC. Thus, in more than 85% of the cases, HCC arises on a cirrhotic liver, regardless of the viral etiology. The size of the tumor was comparable among the 3 groups of patients, with the majority of them presenting a size between 2 and 3 cm, and few patients showed histologically signs of vascular invasion (Additional file 2: Table S2). Among patients in the control group, the liver was either completely normal in 11 individuals (61%) or showed very scanty or mild fatty change in the remaining 7 (39%); all patients but one had normal ALT levels. All were negative for serologic markers of active infection with hepatitis viruses (HBV, HCV, and HDV).

### LncRNA expression profile in HBV-, HCV-, and HDV-related HCC

To investigate if lncRNAs are differentially expressed in HCC of different viral etiology, we first employed the commercial Disease-Related Human LncRNA Profiler, which detects 83 lncRNAs implicated in a variety of human diseases, ranging from neurodegeneration to cancer, including 7 lncRNAs previously associated with HCC (ANRIL, H19, HOTAIR, HOTTIP, HULC, MALAT1 and MEG3). A total of 50 liver specimens obtained from patients with HBV-, HCV- or HDV-associated HCC were analyzed (Fig. 1a).

Among the 83 lncRNAs tested, 15 were not detectable in any liver samples, including HOTAIR, which was previously reported to be associated with HCC [33]. The remaining 68 disease-related lncRNAs were used to investigate the relationship among the six groups of liver specimens: HBV-, HCV- and HDV-related HCC tissues and their surrounding non-tumorous tissues. Principal component analysis of the 68 lncRNA profiles showed a marked separation between tumor and non-tumorous tissues (Fig. 1b), whereas no apparent differences were observed among tumors of different viral etiology (Fig. 1c).

### Identification of lncRNAs dysregulated in HBV-, HCV-, and HDV-related HCC

To identify lncRNAs that were differentially expressed in HBV-, HCV-, and HDV-related HCC, pairwise *t*-test comparisons between each hepatitis virus-associated HCC and paired adjacent non-tumorous tissues were performed on the 68 disease-related lncRNAs. We identified 17 lncRNAs that were dysregulated in at least one hepatitis virus-associated HCC (Additional file 3: Table S3). To confirm our profiling data, we measured the expression levels of these 17 lncRNAs by qRT-PCR using different sets of primers in liver specimens obtained from all 25 patients with HCC, as well as from 20 patients with HBV-, HCV-, or HDV-associated cirrhosis without HCC and in control liver specimens obtained from 10 liver donors and 8 liver resections for hemangioma (Fig. 2a; Additional file 4: Table S4). The majority (14 out of 17) of these lncRNAs were confirmed to be dysregulated in HCC compared to the surrounding non-tumorous tissues (Additional file 5: Table S5**),** corroborating the reliability of our profiling data. To increase the stringency of our analysis, we focused on lncRNAs whose expression was at least twofold up- or down-regulated in tumor tissues compared to the surrounding non-tumorous tissues. Using this criterion, 10 lncRNAs were selected (Fig. 2b). The hierarchical clustering of these 10 lncRNAs and the associated heat map show a separation between matched tumor and non-tumorous tissues, cirrhosis, and normal livers, indicating that most of the identified lncRNAs were selectively dysregulated in tumor samples. One of these lncRNAs, ANRIL, which displayed the highest expression in tumor tissues, was also up-regulated in non-tumorous tissues and in cirrhotic livers without HCC when compared to normal livers (Additional file 4: Table S4). Two of the selected 10 lncRNAs (ANRIL and HOTTIP) have previously been reported to be associated with HCC [34, 35]. Besides ANRIL and HOTTIP, none of the other 8 lncRNAs with >twofold dysregulation was previously identified in HCC studies (Table 1). Two lncRNAs (BC017743 and BC043430) were found to be up-regulated and 6 (aHIF, LINC01152, PAR5, PCAT-29, TMEVPG1, and Y3) down-regulated in HCC tissues compared to paired adjacent non-tumorous tissues (Fig. 2c; Table 1).

**Table 1:** Novel lncRNAs associated with HCC

LncRNA	*P* value			Fold change		
	HBV HCC vs. NT	HCV HCC vs. NT	HDV HCC vs. NT	HBV HCC vs. NT	HCV HCC vs. NT	HDV HCC vs. NT
aHIF	0.760	*0.001*	0.066	+1.159	*−2.594*	−2.707
BC017743	*0.005*	*0.010*	*0.031*	*+9.279*	*+8.362*	*+7.774*
BC043430	*0.006*	*0.014*	*0.024*	*+11.562*	*+9.895*	*+8.551*
LINC01152	*0.038*	*0.003*	*0.041*	*−2.596*	*−4.453*	*−12.191*
PAR5	0.580	*0.005*	0.121	−1.123	*−2.074*	−1.518
PCAT-29	*0.004*	0.343	0.515	*−2.256*	+2.454	−2.012
TMEVPG1	0.099	*0.005*	*0.036*	−4.318	*−5.424*	*−5.108*
Y3	0.198	0.653	*0.045*	−1.419	+1.077	*−2.052*

Statistically significant *P* values were described in italic

*P* values were calculated by Student’s paired *t*-test. The positive sign (+) indicates upregulation of lncRNA expression in HCC; the negative sign (−) indicates downregulation of lncRNA expression in HCC

*HCC* denotes hepatocellular carcinoma, *NT* surrounding nontumorous tissue, *HBV* hepatitis B virus, *HCV* hepatitis C virus, *HDV* hepatitis D virus

Besides the 17 lncRNAs identified from our profiling data, we also measured the expression levels of 18 recently reported HCC-associated lncRNAs that were not included in our profiling analysis by qRT-PCR, using the same primers as previously reported [15–32] (Additional file 6: Table S6). In our study, only 7 of these lncRNAs (DBH-AS1, HEIH, hDREH, PCNA-AS1, hPVT1, UFC1 and ZEB1-AS1) were found to be significantly dysregulated in at least one hepatitis virus-associated HCC (Additional file 7: Table S7).

### LncRNAs shared by HCC regardless of the viral etiology

**Fig. 3:**
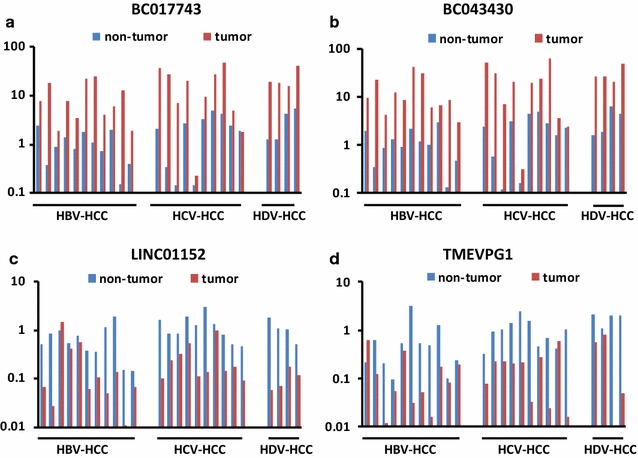
Identification of novel lncRNAs dysregulated in HCC. Expression of lncRNAs in tumor and surrounding non-tumorous tissue in a series of patients with HCC-associated with HBV, HCV and HDV was analyzed by qRT-PCR. The expression levels of lncRNAs was normalized to the house keeping gene GAPDH. The *Y axis* indicates the relative expression of lncRNAs, calculated as described in the “Methods” section. **a** BC017743; **b** BC043430; **c** LINC01152; **d** TMEVPG1

Among the 8 newly identified HCC-related lncRNAs, BC017743 and BC043430 were highly expressed in all but one patient with HCC (24 out of 25; 96%) (Fig. 3a, b), with a fold change around 10 in each viral etiology (Table 1). Likewise, among the down-regulated lncRNAs, LINC01152 expression was reduced in all but one patient with HCC (24 out of 25, 96%) (Fig. 3c), and TMEVPG1 in all but two (23 out of 25, 92%) (Fig. 3d), although the difference did not reach statistical significance in HBV-related HCC (*P* = 0.099) (Table 1).

Among the lncRNAs previously reported in HCC, we found that 6 (ANRIL, HEIH, HOTTIP, PCNA-AS1, UFC1 and ZEB-AS1) were elevated in all three hepatitis viruses-related tumor tissues (Additional file 7: Table S7, Additional file 8: Figure S1a, b, f–i), although some of them did not reach statistical significance, possibly due to the small number of samples analyzed.

### LncRNAs unique to HCC associated with a specific hepatitis virus

**Fig. 4:**
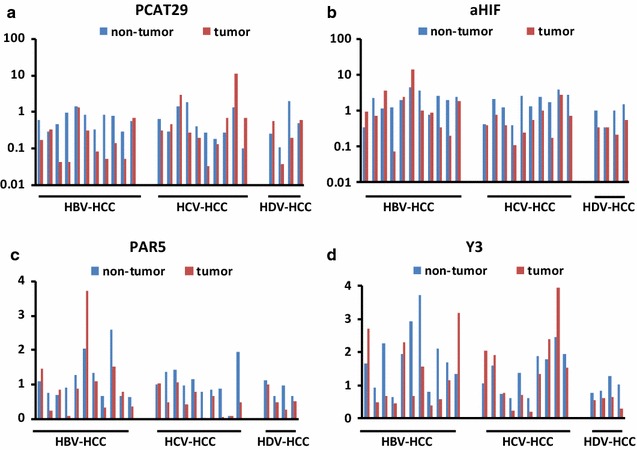
Identification of significantly dysregulated lncRNAs unique to HCC associated with a specific hepatitis virus. The expression of lncRNAs in the tumor and surrounding non-tumorous tissue in a series of patients with HCC-associated with HBV, HCV and HDV was analyzed by qRT-PCR. The expression levels of lncRNAs was normalized to the house keeping gene GAPDH. The Y axis indicates the relative expression of lncRNAs, calculated as described in the “Methods” section. **a** PCAT29; **b** aHIF; **c** PAR5; **d** Y3

Next, we investigated whether some lncRNAs were specifically associated with a single hepatitis virus etiology. Interestingly, PCAT-29 was dysregulated predominantly in HBV-related HCC, aHIF and PAR5 in HCV-related HCC, and Y3 in HDV-related HCC (Fig. 2c). PCAT-29 was down-regulated in tumor vs. the surrounding non-tumorous tissue of 8 out of 11 (73%) HBV-related HCC patients (*P* = 0.004) (Fig. 4a; Table 1). Two lncRNAs, aHIF and PAR5, were significantly down-regulated in HCV-related HCC tissues (9 out of 10, 90% for both; *P* = 0.001 and *P* = 0.005, respectively) (Fig. 4b, c; Table 1). Overexpression of Y3, which was previously reported in solid tumors, e.g. bladder, cervix, colon carcinoma [36], was found in only 3 of 11 HBV-related HCC samples and 4 of 10 HCV-related HCC samples (Fig. 4d). Y3 was down-regulated in HDV-related tumor tissues compared to the surrounding non-tumorous tissues (*P* = 0.045) (Fig. 4d; Table 1).

Among the lncRNAs previously reported in HCC, we found that three lncRNAs, DBH-AS1, hDREH and hPVT1, behaved differently according to the hepatitis virus involved in HCC (Additional file 9: Figure S2a–c). DBH-AS1 was found to be down-regulated in all HBV-related HCC samples (11 out of 11, 100%; *P* < 0.001) compared with adjacent non-tumor liver tissues (Additional file 7: Table S7, Additional file 9: Figure S2a). In contrast, this lncRNA was not significantly dysregulated in HCV- and HDV-related HCC tissues (Additional file 7: Table S7, Additional file 9: Figure S2a). Expression of hDREH was frequently reduced (8 out of 11, 73%) in HBV-related HCC (Additional file 9: Figure S2b), as reported in a previous study [19], although this trend did not reach statistical significance (*P* = 0.052) (Additional file 7: Table S7). On the contrary, hDREH was up-regulated in most cases of HCV-associated HCC (9 out of 10, 90%; *P* = 0.007) (Additional file 7: Table S7, Additional file 9: Figure S2b). Regarding hPVT1, we found that it was up-regulated in our HBV-related HCC patients (10 out of 11, 91%; *P* = 0.004) (Additional file 7: Table S7, Additional file 9: Figure S2c). By contrast, the difference did not reach statistical significance in HCV-, and HDV-related HCC tissues (Additional file 7: Table S7, Additional file 9: Figure S2c).

## Discussion

To the best of our knowledge, this is the first study in which the expression of lncRNAs was analyzed in paired tumorous and nontumorous liver specimens obtained from patients with HBV-, HCV-, and HDV-associated HCC. Although the number of patients that could be included in this comprehensive study was limited, our patients were well characterized in terms of clinical, virologic and pathological features, and devoid of confounding factors. It is estimated that 7000–23,000 lncRNAs exist in the human genome, with approximately 6700 already identified [1]. However, only about 180 human lncRNAs recorded in the lncRNAdb (the reference database for functional long noncoding RNAs) have been investigated and proved to be biologically functional [37]. In our study, we profiled 101 lncRNAs, which comprise 83 lncRNAs included in Disease-Related Human LncRNA profiler and an additional 18 lncRNAs that have recently been associated with HCC [15–32]. Compared to previous studies of lncRNAs in HCC, which were mostly performed in HBV-related HCC, we performed lncRNAs expression profiling in HCC associated with all three hepatitis viruses that cause chronic infection with the aim of investigating whether certain lncRNAs are selectively dysregulated according to the different viral etiology of HCC. Moreover, our study also included tissues from normal liver and cirrhosis without HCC, while most of the other studies only investigated lncRNAs in HCC patients.

Access to a unique collection of paired liver samples from patients with HCC of different etiology allowed us to identify 8 lncRNAs that have not been previously associated with HCC. Six of these lncRNAs, namely, aHIF, BC017743, BC043430, PAR5, PCAT29 and Y3, have already been found to be dysregulated in other tumors [36, 38–41]. Except for Y3, the expression of five other cancer-related lncRNAs in HCC showed the same trend as previously reported in other tumors. aHIF was previously found to be down-regulated in breast cancer, where it can serve as a prognostic marker [38]. BC017743 and BC043430 are both located in the tumor suppressor region at 3p12.3. Dysregulation of BC017743 and BC043430 has been found in lung, breast and kidney cancers, but the function of these lncRNAs remains unclear [39]. Low expressions of PAR5 and PCAT29 were correlated with poor prognostic outcomes in human glioblastoma multiforme and prostate cancer, respectively [40, 41]. Only PCAT29 has been shown to function as a tumor suppressor [41], while the functions of PAR5 have not been investigated. Overexpression of Y3 was found in bladder, cervix, colon, kidney, lung and prostate cancer where it seems to be required for cell proliferation [36]. However, we found that Y3 was significantly down-regulated in HDV-related HCC, indicating a differential role of Y3 in the development of different cancers. Interestingly, our study provides the first evidence for an association between the remaining two lncRNAs, LINC01152 and TMEVPG1, and cancer, specifically HCC. LINC01152 was first identified in a patient with campomelic dysplasia, and its biological function remains to be fully elucidated [42]. We found that LINC01152 was also expressed in the liver and was frequently down-regulated in HCC tissues, suggesting that it may play a role as a tumor suppressor. TMEVPG1 was found within the Tmevpg3 genetic locus which controls Theiler’s virus persistence [43]. We found its upregulation seems to be related to liver cirrhosis, since no difference was observed between tumor and normal livers (Additional file 4: Table S4).

When we analyzed whether there was any relationship between these 8 lncRNAs and a specific hepatitis virus associated with HCC, we found that 4 lncRNAs, BC017743, BC043430, LINC01152 and TMEVPG1, were highly up- or down-regulated in hepatitis viruses-related HCC. Interestingly, the other 4 lncRNAs, PCAT-29, aHIF, PAR5 and Y3, were significantly down-regulated predominantly in one specific hepatitis virus-related HCC: PCAT-29 in HBV-related HCC, aHIF and PAR5 in HCV-related HCC, and Y3 in HDV-related HCC. This suggests that HCCs of different viral etiologies are regulated, at least in part, by different lncRNAs. Since there is very limited information about these lncRNAs, additional studies will be needed to elucidate the mechanistic connection between these lncRNAs and the molecular pathogenesis of HCC associated with different hepatitis viruses. However, the identification of the lncRNA related to specific hepatitis virus-related HCC might be useful as accurate diagnostic markers for HCC of different viral etiology.

The Disease-Related Human LncRNA Profiler we employed in our study also includes several lncRNAs that were previously associated with HCC [34, 35, 44–46]. Our data are generally consistent with previous reports [34, 35, 44, 46]. For example, we found that ANRIL and HOTTIP expression levels were increased, while H19 and MEG3 expression levels were decreased, although these changes did not reach statistical significance in HCC tissues due to dramatically elevated expression of these two lncRNAs in some tumor tissues (Additional file 8: Figure S1 c, e), as also reported in other studies [44, 46]. Our findings show that these lncRNAs are commonly dysregulated in all hepatitis virus-associated HCC tissues, indicating that their functions in HCC are not associated with any specific hepatitis virus. In contrast to HOTTIP, H19 and MEG3, we found that ANRIL expression was up-regulated both in cirrhosis vs. normal liver and in HCC vs. cirrhosis, suggesting its involvement in the process of hepatocarcinogenesis from normal liver through the precancerous stage of cirrhosis. ANRIL is the antisense RNA of the tumor suppressor gene p15/CDKN2A, which can epigenetically silence p15/CDKN2A in *cis* and in *trans* through heterochromatin formation or DNA methylation [47, 48]. It is likely that ANRIL functions in HCC progression through the same mechanism, since the p15 promoter is frequently methylated in tumor tissues from HCC patients [49]. HULC, which was identified as the first hepatocyte-specific lncRNA, was reported to be highly up-regulated in HCC [45, 50]. However, our data showed that changes in its expression were not statistically significant, being either up- or down-regulated in HCC tissues (Additional file 8: Figure S1d), indicating that the role of HULC in HCC might be more complex and requires further investigation.

Besides these previously reported HCC-related lncRNAs included in the profiler, more than 10 additional lncRNAs have been associated in HCC studies in the past 5 years, which were mostly performed in HBV-related HCC [51]. In our analysis, three lncRNAs, DBH-AS1, hDREH and hPVT1, were differentially expressed according to the viral etiology in HCC. Among these, both DBH-AS1 and hDREH are regulated by the HBV HBx protein [18, 19]. Thus, it is reasonable that they showed different expression patterns in HBV-related HCC compared to HCV-related HCC. However, we also found something unexpected. In a previous report, the expression of DBH-AS1 was only analyzed in HCC tissues, and experimental evidence showed that this lncRNA promotes cell proliferation and survival by activating MAPK signaling in HCC [18]. In our study, we found that DBH-AS1 was down-regulated in all of our HBV-related HCC tissues compared to surrounding non-tumorous tissues, which appears to contradict its potential oncogenic role in HCC [18]. The lncRNA Dreh was previously found to be inhibited by HBx protein in mice and to act as a tumor suppressor in HBV-related HCC [19]. hDREH, the human homolog of Dreh, was significantly down-regulated in HBV-related HCC tissues compared to the surrounding non-tumorous tissues [19]. However, we found that hDREH was up-regulated in HCV-related HCC, which is contrary to what we and others have observed in HBV-related HCC, suggesting the existence of other factors that may affect hDREH expression besides HBx. Although HDV-related HCC patients were co-infected with HBV and HDV, HBV replication was reduced about 100-fold compared to HBV-monoinfected cases [52], which might explain why DBH-AS1 and hDREH were not as significantly dysregulated in these patients as in HBV-related HCC patients. The oncofetal lncRNA hPVT1 was found to promote proliferation and adoption of stem cell-like properties by HCC cells [27]. A previous report also showed that hPVT1 was up-regulated through the TGF-β pathway, which can be activated by HBV infection in HBV-associated HCC tissues [53]. Our data supports this conclusion by showing that hPVT1 was significantly up-regulated only in HBV-related HCC, not in HCV- and HDV-related HCC.

Several recently reported HCC-related lncRNAs were not significantly dysregulated in HCC tissues in our study. Besides the relatively limited sample size of our series, there are several other possible explanations for these findings. First, the difference between lncRNAs expression in tumor tissues compared to non-tumorous tissues in some instances, e.g. MVIH and LET [22, 25], was marginal (<onefold), which could be easily missed due to technical issues. Second, the method we used to detect some lncRNAs is different from those reported in other studies. For example, uc.338 expression was investigated in HCC tissues by in situ hybridization [28], while in our study we used qRT-PCR.

## Conclusions

Our study provides new insights about the expression and potential role of lncRNAs in HCC. Since lncRNAs are emerging as key regulators of many cellular functions, it will be important to investigate the role of these lncRNAs in HCC progression and their potential usefulness as diagnostic tools or therapeutic targets for HCC.

## Additional files

**Additional file 1: Table S1.** Primer sequences used in our study.

**Additional file 2: Table S2.** Clinical characteristics of 25 HCC patients and 20 cirrhotic patients.

**Additional file 3: Table S3.** Seventeen lncRNAs significantly dysregulated on profiling data in HCC patients.

**Additional file 4: Table S4.** Relative expression levels of seventeen dysregulated lncRNAs in HCC patients.

**Additional file 5: Table S5.** Eighteen lncRNAs previously reported to be associated with HCC.

**Additional file 6: Table S6.** Relative expression levels of eighteen previously identified HCC-associated lncRNAs.

**Additional file 7: Table S7.** Eighteen lncRNAs previously reported to be associated with HCC.

**Additional file 8: Figure S1.** Expression of HCC-associated lncRNAs in the tumor and surrounding non-tumorous tissue in a series of patients with HCC associated with HBV, HCV and HDV analyzed by qRT-PCR.

**Additional file 9: Figure S2.** The lncRNAs, DBH-AS1, hDREH and hPVT1 were differentially dysregulated in HBV-, HCV-, and HDV-related HCC.

## Abbreviations

ALT: alanine aminotransferase
ANOVA: analysis of variance
HBV: hepatitis B virus
HBx: hepatitis B virus x protein
HCC: hepatocellular carcinoma
HCV: hepatitis C virus
HDV: hepatitis D virus
lncRNAs: long noncoding RNAs
MAPK: mitogen-activated protein kinase
qRT-PCR: quantitative real-time polymerase chain reaction
sncRNAs: small non-coding RNAs
TGF-β: transforming growth factor beta

## References

[1] Zhang Q, Jeang KT (2013). Long non-coding RNAs (lncRNAs) and viral infections. Biomed Pharmacother.

[2] Zhao J, Greene CM, Gray SG, Lawless MW (2014). Long noncoding RNAs in liver cancer: what we know in 2014. Expert Opin Ther Targets..

[3] Prensner JR, Chinnaiyan AM (2011). The emergence of lncRNAs in cancer biology. Cancer Discov..

[4] Li X, Wu Z, Fu X, Han W (2013). Long noncoding RNAs: insights from biological features and functions to diseases. Med Res Rev.

[5] Wallace MC, Preen D, Jeffrey GP, Adams LA (2015). The evolving epidemiology of hepatocellular carcinoma: a global perspective. Expert Rev Gastroenterol Hepatol..

[6] Nordenstedt H, White DL, El-Serag HB (2010). The changing pattern of epidemiology in hepatocellular carcinoma. Dig Liver Dis..

[7] Schweitzer A, Horn J, Mikolajczyk RT, Krause G, Ott JJ (2015). Estimations of worldwide prevalence of chronic hepatitis B virus infection: a systematic review of data published between 1965 and 2013. Lancet.

[8] Thomas DL (2013). Global control of hepatitis C: where challenge meets opportunity. Nat Med.

[9] Alvarado-Mora MV, Locarnini S, Rizzetto M, Pinho JR (2013). An update on HDV: virology, pathogenesis and treatment. Antivir Ther..

[10] Guidotti LG, Chisari FV (2006). Immunobiology and pathogenesis of viral hepatitis. Annu Rev Pathol.

[11] Braconi C, Patel T (2012). Non-coding RNAs as therapeutic targets in hepatocellular cancer. Curr Cancer Drug Targets.

[12] Takahashi K, Yan I, Haga H, Patel T (2014). Long noncoding RNA in liver diseases. Hepatology.

[13] Ishak K, Baptista A, Bianchi L, Callea F, De Groote J, Gudat F, Denk H, Desmet V, Korb G, MacSween RN (1995). Histological grading and staging of chronic hepatitis. J Hepatol.

[14] Edmondson HA, Steiner PE (1954). Primary carcinoma of the liver: a study of 100 cases among 48,900 necropsies. Cancer.

[15] Lu X, Zhou C, Li R, Liang Z, Zhai W, Zhao L, Zhang S (2016). Critical role for the long non-coding RNA AFAP1-AS1 in the proliferation and metastasis of hepatocellular carcinoma. Tumour Biol..

[16] Deng L, Yang SB, Xu FF, Zhang JH (2015). Long noncoding RNA CCAT1 promotes hepatocellular carcinoma progression by functioning as let-7 sponge. J Exp Clin Cancer Res..

[17] Yuan SX, Wang J, Yang F, Tao QF, Zhang J, Wang LL, Yang Y, Liu H, Wang ZG, Xu QG (2016). Long noncoding RNA DANCR increases stemness features of hepatocellular carcinoma by derepression of CTNNB1. Hepatology.

[18] Huang JL, Ren TY, Cao SW, Zheng SH, Hu XM, Hu YW, Lin L, Chen J, Zheng L, Wang Q (2015). HBx-related long non-coding RNA DBH-AS1 promotes cell proliferation and survival by activating MAPK signaling in hepatocellular carcinoma. Oncotarget..

[19] Huang JF, Guo YJ, Zhao CX, Yuan SX, Wang Y, Tang GN, Zhou WP, Sun SH (2013). Hepatitis B virus X protein (HBx)-related long noncoding RNA (lncRNA) down-regulated expression by HBx (Dreh) inhibits hepatocellular carcinoma metastasis by targeting the intermediate filament protein vimentin. Hepatology.

[20] Chang L, Li C, Lan T, Wu L, Yuan Y, Liu Q, Liu Z (2016). Decreased expression of long non-coding RNA GAS5 indicates a poor prognosis and promotes cell proliferation and invasion in hepatocellular carcinoma by regulating vimentin. Mol Med Rep..

[21] Yang F, Zhang L, Huo XS, Yuan JH, Xu D, Yuan SX, Zhu N, Zhou WP, Yang GS, Wang YZ (2011). Long noncoding RNA high expression in hepatocellular carcinoma facilitates tumor growth through enhancer of zeste homolog 2 in humans. Hepatology.

[22] Yang F, Huo XS, Yuan SX, Zhang L, Zhou WP, Wang F, Sun SH (2013). Repression of the long noncoding RNA-LET by histone deacetylase 3 contributes to hypoxia-mediated metastasis. Mol Cell.

[23] Ji J, Tang J, Deng L, Xie Y, Jiang R, Li G, Sun B (2015). LINC00152 promotes proliferation in hepatocellular carcinoma by targeting EpCAM via the mTOR signaling pathway. Oncotarget..

[24] Wang Y, He L, Du Y, Zhu P, Huang G, Luo J, Yan X, Ye B, Li C, Xia P (2015). The long noncoding RNA lncTCF7 promotes self-renewal of human liver cancer stem cells through activation of Wnt signaling. Cell Stem Cell.

[25] Yuan SX, Yang F, Yang Y, Tao QF, Zhang J, Huang G, Yang Y, Wang RY, Yang S, Huo XS (2012). Long noncoding RNA associated with microvascular invasion in hepatocellular carcinoma promotes angiogenesis and serves as a predictor for hepatocellular carcinoma patients’ poor recurrence-free survival after hepatectomy. Hepatology.

[26] Yuan SX, Tao QF, Wang J, Yang F, Liu L, Wang LL, Zhang J, Yang Y, Liu H, Wang F (2014). Antisense long non-coding RNA PCNA-AS1 promotes tumor growth by regulating proliferating cell nuclear antigen in hepatocellular carcinoma. Cancer Lett.

[27] Wang F, Yuan JH, Wang SB, Yang F, Yuan SX, Ye C, Yang N, Zhou WP, Li WL, Li W (2014). Oncofetal long noncoding RNA PVT1 promotes proliferation and stem cell-like property of hepatocellular carcinoma cells by stabilizing NOP2. Hepatology.

[28] Braconi C, Valeri N, Kogure T, Gasparini P, Huang N, Nuovo GJ, Terracciano L, Croce CM, Patel T (2011). Expression and functional role of a transcribed noncoding RNA with an ultraconserved element in hepatocellular carcinoma. Proc Natl Acad Sci USA.

[29] Wang F, Ying HQ, He BS, Pan YQ, Deng QW, Sun HL, Chen J, Liu X, Wang SK (2015). Upregulated lncRNA-UCA1 contributes to progression of hepatocellular carcinoma through inhibition of miR-216b and activation of FGFR1/ERK signaling pathway. Oncotarget..

[30] Cao C, Sun J, Zhang D, Guo X, Xie L, Li X, Wu D, Liu L (2015). The long intergenic noncoding RNA UFC1, a target of MicroRNA 34a, interacts with the mRNA stabilizing protein HuR to increase levels of beta-catenin in HCC cells. Gastroenterology.

[31] Li T, Xie J, Shen C, Cheng D, Shi Y, Wu Z, Deng X, Chen H, Shen B, Peng C (2016). Upregulation of long noncoding RNA ZEB1-AS1 promotes tumor metastasis and predicts poor prognosis in hepatocellular carcinoma. Oncogene.

[32] Li T, Xie J, Shen C, Cheng D, Shi Y, Wu Z, Deng X, Chen H, Shen B, Peng C (2015). Amplification of long noncoding RNA ZFAS1 promotes metastasis in hepatocellular carcinoma. Cancer Res.

[33] Geng YJ, Xie SL, Li Q, Ma J, Wang GY (2011). Large intervening non-coding RNA HOTAIR is associated with hepatocellular carcinoma progression. J Int Med Res.

[34] Quagliata L, Matter MS, Piscuoglio S, Arabi L, Ruiz C, Procino A, Kovac M, Moretti F, Makowska Z, Boldanova T (2014). Long noncoding RNA HOTTIP/HOXA13 expression is associated with disease progression and predicts outcome in hepatocellular carcinoma patients. Hepatology.

[35] Hua L, Wang CY, Yao KH, Chen JT, Zhang JJ, Ma WL (2015). High expression of long non-coding RNA ANRIL is associated with poor prognosis in hepatocellular carcinoma. Int J Clin Exp Pathol..

[36] Christov CP, Trivier E, Krude T (2008). Noncoding human Y RNAs are overexpressed in tumours and required for cell proliferation. Br J Cancer.

[37] Quek XC, Thomson DW, Maag JL, Bartonicek N, Signal B, Clark MB, Gloss BS, Dinger ME (2015). lncRNAdb v2.0: expanding the reference database for functional long noncoding RNAs. Nucleic Acids Res.

[38] Cayre A, Rossignol F, Clottes E, Penault-Llorca F (2003). aHIF but not HIF-1alpha transcript is a poor prognostic marker in human breast cancer. Breast Cancer Res.

[39] Ghosh S, Ghosh A, Maiti GP, Alam N, Roy A, Roychoudhury S, Panda CK (2009). Alterations of ROBO1/DUTT1 and ROBO2 loci in early dysplastic lesions of head and neck: clinical and prognostic implications. Hum Genet.

[40] Zhang XQ, Sun S, Lam KF, Kiang KM, Pu JK, Ho AS, Lui WM, Fung CF, Wong TS, Leung GK (2013). A long non-coding RNA signature in glioblastoma multiforme predicts survival. Neurobiol Dis.

[41] Malik R, Patel L, Prensner JR, Shi Y, Iyer MK, Subramaniyan S, Carley A, Niknafs YS, Sahu A, Han S (2014). The lncRNA PCAT29 inhibits oncogenic phenotypes in prostate cancer. Mol Cancer Res.

[42] Ninomiya S, Isomura M, Narahara K, Seino Y, Nakamura Y (1996). Isolation of a testis-specific cDNA on chromosome 17q from a region adjacent to the breakpoint of t(12;17) observed in a patient with acampomelic campomelic dysplasia and sex reversal. Hum Mol Genet.

[43] Vigneau S, Levillayer F, Crespeau H, Cattolico L, Caudron B, Bihl F, Robert C, Brahic M, Weissenbach J, Bureau JF (2001). Homology between a 173-kb region from mouse chromosome 10, telomeric to the Ifng locus, and human chromosome 12q15. Genomics.

[44] Zhang L, Yang F, Yuan JH, Yuan SX, Zhou WP, Huo XS, Xu D, Bi HS, Wang F, Sun SH (2013). Epigenetic activation of the MiR-200 family contributes to H19-mediated metastasis suppression in hepatocellular carcinoma. Carcinogenesis.

[45] Wang J, Liu X, Wu H, Ni P, Gu Z, Qiao Y, Chen N, Sun F, Fan Q (2010). CREB up-regulates long non-coding RNA, HULC expression through interaction with microRNA-372 in liver cancer. Nucleic Acids Res.

[46] Braconi C, Kogure T, Valeri N, Huang N, Nuovo G, Costinean S, Negrini M, Miotto E, Croce CM, Patel T (2011). microRNA-29 can regulate expression of the long non-coding RNA gene MEG3 in hepatocellular cancer. Oncogene.

[47] Yap KL, Li S, Munoz-Cabello AM, Raguz S, Zeng L, Mujtaba S, Gil J, Walsh MJ, Zhou MM (2010). Molecular interplay of the noncoding RNA ANRIL and methylated histone H3 lysine 27 by polycomb CBX7 in transcriptional silencing of INK4a. Mol Cell.

[48] Yu W, Gius D, Onyango P, Muldoon-Jacobs K, Karp J, Feinberg AP, Cui H (2008). Epigenetic silencing of tumour suppressor gene p15 by its antisense RNA. Nature.

[49] Wong IH, Lo YM, Yeo W, Lau WY, Johnson PJ (2000). Frequent p15 promoter methylation in tumor and peripheral blood from hepatocellular carcinoma patients. Clin Cancer Res.

[50] Panzitt K, Tschernatsch MM, Guelly C, Moustafa T, Stradner M, Strohmaier HM, Buck CR, Denk H, Schroeder R, Trauner M (2007). Characterization of HULC, a novel gene with striking up-regulation in hepatocellular carcinoma, as noncoding RNA. Gastroenterology.

[51] Parasramka MA, Maji S, Matsuda A, Yan IK, Patel T (2016). Long non-coding RNAs as novel targets for therapy in Hepatocellular Carcinoma. Pharmacol Ther.

[52] Pollicino T, Raffa G, Santantonio T, Gaeta GB, Iannello G, Alibrandi A, Squadrito G, Cacciola I, Calvi C, Colucci G (2011). Replicative and transcriptional activities of hepatitis B virus in patients coinfected with hepatitis B and hepatitis delta viruses. J Virol.

[53] Yang P, Li QJ, Feng Y, Zhang Y, Markowitz GJ, Ning S, Deng Y, Zhao J, Jiang S, Yuan Y (2012). TGF-beta-miR-34a-CCL22 signaling-induced Treg cell recruitment promotes venous metastases of HBV-positive hepatocellular carcinoma. Cancer Cell.

