# Supplementary material for: Analysis of long noncoding RNA expression in hepatocellular carcinoma of different viral etiology
Source: J Transl Med. 2016 Nov 28;14:328. doi: 10.1186/s12967-016-1085-4 (PMC5125040; doi:10.1186/s12967-016-1085-4)
Supplement: Supplementary file 1 — Additional file 1: Table S1. Primer sequences used in our study. [file 12967_2016_1085_MOESM1_ESM.docx]

**Table S1. Primer sequences used in our study**

| Gene |  | Sequence |
| --- | --- | --- |
| AFAP1-AS1 | Sense primer | TCGCTCAATGGAGTGACGGCA |
|  | Anti-sense primer | CGGCTGAGACCGCTGAGAACTT |
|  |  |  |
| ANRIL | Sense primer | CAACATCCACCACTGGATCTTAACA |
|  | Anti-sense primer | AGCTTCGTATCCCCAATGAGATACA |
| AK023948 | Sense primer  Anti-sense primer | CAGGGCATGGCTGTGTTT  GACATTATCAAGTAACTAGTGCTGTCA |
| BC017743 | Sense primer  Anti-sense primer | GTTCCCTGTCTTCCTTATTTCCC  GCTCTTCATCACATTTTCCCATCG |
| BC043430 | Sense primer | CTGTACTACAGGGAATCTCTC |
|  | Anti-sense primer | CTTCCTTTGGGTCTGTTCAG |
| CCAT1 | Sense primer | TTTATGCTTGAGCCTTGA |
|  | Anti-sense primer | CTTGCCTGAAATACTTGC |
|  |  |  |
| DANCR | Sense primer | GCGCCACTATGTAGCGGGTT |
|  | Anti-sense primer | TCAATGGCTTGTGCCTGTAGTT |
|  |  |  |
| DBH-AS1 | Sense primer | CGTCCACTCGTCTGTTCACT |
|  | Anti-sense primer | TAACACCCCATCCGCTTGT |
|  |  |  |
| hDREH | Sense primer | GCTAACGAACAAAGCCAGA |
|  | Anti-sense primer | CCCTATTCTCATGCAAGGA |
| GAS5 | Sense primer | CAACTTGCCTGGACCAGCTT |
|  | Anti-sense primer | TCAAGCCGACTCTCCATACC |
| H19 | Sense primer | GCACCTTGGACATCTGGAGT |
|  | Anti-sense primer | TTCTTTCCAGCCCTAGCTCA |
|  |  |  |
| aHIF | Sense primer | TTTGTGTTTGAGCATTTTAATAGGC |
|  | Anti-sense primer | CCAGGCCCCTTTGATCAGCTT |
|  |  |  |
| HEIH | Sense primer | CCTCTTGTGCCCCTTTCTT |
|  | Anti-sense primer | ATGGCTTCTCGCATCCTAT |
| HOTTIP | Sense primer | CAGCGACCCGTCTTGTAG |
|  | Anti-sense primer | TGTGCCCACTTTCACTTTG |
| HULC | Sense primer | ATCTGCAAGCCAGGAAGAGTC |
|  | Anti-sense primer | CTTGCTTGATGCTTTGGTCTGT |
| IPW | Sense primer  Anti-sense primer | TGCCTAGACCACCCACTAAAGG  AGTCTCCATGCGGAAGGAAGA |
|  |  |  |
| LET | Sense primer | CCTTCCTGACAGCCAGTGTG |
|  | Anti-sense primer | CAGAATGGAAATACTGGAGCAAG |
| Linc00152 | Sense primer | CTCCAGCACCTCTACCTGTTG |
|  | Anti-sense primer | GGACAAGGGATTAAGACACACA |
| Linc00974 | Sense primer | TCTAACGTGCCTGGGACCTA |
|  | Anti-sense primer | AAATGCCTACCGCCAGTTCA |
|  |  |  |
| LINC01152 | Sense primer | CAGGTCCTTGGTCTCACG |
|  | Anti-sense primer | TTCCCATTGGATTGTTGT |
| LincTCF7 | Sense primer | AGGAGTCCTTGGACCTGAGC |
|  | Anti-sense primer | AGTGGCTGGCATATAACCAACA |
|  |  |  |
| MEG3 | Sense primer | CATCCTGCTGGCAACTCC |
|  | Anti-sense primer | TTCCCCCAGAAAAGGATAGG |
|  |  |  |
| MVIH | Sense primer | GAGACAGGATTTAGCCGTGTTG |
|  | Anti-sense primer | AGCACTTTGGAAGGCTTAGACA |
|  |  |  |
| PCAT29 | Sense primer | ATCGGCATGTGACTTGATT |
|  | Anti-sense primer | CATCTCCCACCTCTGCTCTC |
|  |  |  |
| PCNA-AS1 | Sense primer | GTCCTTGAGTGCCTCCAACAC |
|  | Anti-sense primer | ACCAGCTAGACTTTCCTCCTTCC |
|  |  |  |
| PTENP1 | Sense primer | AGTCACCTGTTAAGAAAATGAGAAGACAAA |
|  | Anti-sense primer | CTGTCCCTTATCAGATACATGACTTTCAA |
|  |  |  |
| hPVT1 | Sense primer | AAAACGGCAGCAGGAAATGT |
|  | Anti-sense primer | ATTCCCATAGAAGGGGCAGG |
|  |  |  |
| PAR5 | Sense primer | TGATGTGGGTGTTGATAC |
|  | Anti-sense primer | ACTCAAAGGCAAGAACTA |
|  |  |  |
| ST7OT1 | Sense primer | TCCCTACAAGTGGCTTTCGT |
|  | Anti-sense primer | CCTAGCTGTCCCGGGTTTAT |
|  |  |  |
| TMEVPG1 | Sense primer | GAAGGAATAAGCCTGGAAGA |
|  | Anti-sense primer | TACCATGTTGTCTTAGCTGC |
|  |  |  |
| uc.338 | Sense primer | AGCGACAGTGCGAGCTTT |
|  | Anti-sense primer | GGAAGGATTGAGTGAGCCTT |
|  |  |  |
| UCA1 | Sense primer | CTCTCCTATCTCCCTTCACTGA |
|  | Anti-sense primer | CTTTGGGTTGAGGTTCCTGT |
|  |  |  |
| UFC1 | Sense primer | TCCAACCTGAGTGACATAGCGA |
|  | Anti-sense primer | CTGACCTCCAACTCCAACGAAT |
|  |  |  |
| Y3 | Sense primer | GGCTGGTCCGAGTGCAGTGG |
|  | Anti-sense primer | GAAGCAGTGGGAGTGGAGAA |
|  |  |  |
| ZEB-AS1 | Sense primer | CCGTGGGCACTGCTGAAT |
|  | Anti-sense primer | CTGCTGGCAAGCGGAACT |
|  |  |  |
| ZFAS1 | Sense primer | ACGTGCAGACATCTACAACCT |
|  | Anti-sense primer | TACTTCCAACACCCGCAT |
|  |  |  |
| 7SK | Sense primer | CTTCGGTCAAGGGTATACGAGT |
|  | Anti-sense primer | ATGCAGCGCCTCATTTGGATGT |
|  |  |  |
| GAPDH | Sense primer | CTCTGCTCCTCCTGTTCGAC |
|  | Anti-sense primer | TTAAAAGCAGCCCTGGTGAC |
|  |  |  |
