# Supplementary material for: Analysis of long noncoding RNA expression in hepatocellular carcinoma of different viral etiology
Source: J Transl Med. 2016 Nov 28;14:328. doi: 10.1186/s12967-016-1085-4 (PMC5125040; doi:10.1186/s12967-016-1085-4)
Supplement: Supplementary file 2 — Additional file 2: Table S2. Clinical characteristics of 25 HCC patients and 20 cirrhotic patients. [file 12967_2016_1085_MOESM2_ESM.docx]

**Table S2. Clinical characteristics of 25 HCC patients and 20 cirrhotic patients**

| **Characteristic** | **HBV** | **HCV** | | | | | **HDV** |  | | | |  | |  | | |  |
| --- | --- | --- | --- | --- | --- | --- | --- | --- | --- | --- | --- | --- | --- | --- | --- | --- | --- |
| **HCC** n=11 n=10 n=4 | | | | | | | | | | | | | | |  |  |  |
| Age, *yr* | 60±8 | | | 55±11 | 59±7 | | | |  | | | |  | | | | |
| Male, No.(%) | 10 (91) | | | 9 (90) | 4 (100) | | | |  | | | |  | | | | |
| Platelets (10^3^/µL) | 154±94 | | | 177±181 | 87±18 | | | |  | | | |  | | | | |
| Alanine aminostransferase (*IU/L*)* | 36±19 | | | 89±24 | 65±28 | | | |  | | | |  | | | | |
| Aspartate aminotransferase (*IU/L*)† | 40±19 | | | 129±90 | 84±54 | | | |  | | | |  | | | | |
| γ-glutamyltransferase (*U/L^)^*§ | 94±84 | | | 245±155 | 107±50 | | | |  | | | |  | | | | |
| Total bilirubin (*mg/dL^)^* ¶ | 0.9±0.5 | | | 4.2±2.4 | 2.9±3.0 | | | |  | | | |  | | | | |
| Albumin (g/dL) | 3.9±0.6 | | | 3.1±0.3 | 3.3±0.4 | | | |  | | | |  | | | | |
| α-fetoprotein (*ng/mL)* ‡ | 36±90 | | | 20±7 | 20±26 | | | |  | | | |  | | | | |
| Non-tumorous tissue |  | | |  |  | | | |  | | | |  | | | | |
| Fibrosis Stage | 5±2 | | | 6±1 | 6±0 | | | |  | | | |  | | | | |
| F5/F6, No. | 9 | | | 9 | 4 | | | |  | | | |  | | | | |
| Tumor Grade |  | | |  |  | | | |  | | | |  | | | | |
| G2, No. | 7 | | | 5 | 1 | | | |  | |  | |  |  |  |  |  |
| G3, No. | 3 | | | 5 | 3 | | | |  | |  | |  |  |  |  |  |
| G4, No. | 1 | | | 0 | 0 | | | |  | |  | |  |  |  |  |  |
| Tumor size |  | | |  |  | | | |  | |  | |  |  |  |  |  |
| ≥2 and ≤ 3 cm, No. | 8 | | | 6 | 2 | | | |  | |  | |  |  |  |  |  |
| >3 cm, No. | 3 | | | 4 | 2 | | | |  | |  | |  |  |  |  |  |
| Vascular invasion, No. | 4 | | | 2 | 0 | | | |  | |  | |  |  |  |  |  |
| **Cirrhosis** n=3 n=10 n=7 | | | | | | | | | | | | | | |  |  |  |
| Age, *yr* | 52±6 | | 49±8 | | | 56±3 | | | |  | |  | | | |  |  |
| Male, No.(%) | 2 (67) | | 9 (90) | | | 4 (57) | | | |  | |  | | | |  |  |
| Platelets (10^3^/µL) | 52±16 | | 79±35 | | | 54±30 | | | |  | |  | | | |  |  |
| Alanine aminostransferase (*U/L)** | 23±1 | | 80±51 | | | 78±61 | | | |  | |  | | | |  |  |
| Aspartate aminotransferase (*U/L*)† | 39±10 | | 132±117 | | | 81±37 | | | |  | |  | | | |  |  |
| γ-glutamyltransferase (*U/L^)^*§ | 39±27 | | 51±36 | | | 67±52 | | | |  | |  | | | |  |  |
| Total bilirubin (*mg/dL)*¶ | 2.8±1.6 | | 1.1±0.7 | | | 2.4±1.0 | | | |  | |  | | | |  |  |
| Albumin, (g/dL) | 3.7±0.4 | | 3.0±0.5 | | | 3.6±0.6 | | | |  | |  | | | |  |  |
| α-fetoprotein (*ng/mL)* ‡ | 6±8 | | 47±62 | | | 13±16 | | | |  | |  | | | |  |  |
| Fibrosis Stage | 6±0 | | 6±0 | | | 6±0 | | | |  | |  | | | |  |  |

HCC denotes hepatocellular carcinoma; HBV, hepatitis B virus; HCV, hepatitis C virus;

HDV, hepatitis D virus. Data are expressed as number (%) or the mean ± standard deviation.

* Normal values for alanine aminotransferase range from 3 to 30 units per liter.

† Normal values for aspartate aminotransferase range from 5 to 34 units per liter.

§ Normal values for γ-glutamyltransferase range from 7 to 38 units per liter

¶ To convert the values for total bilirubin to µmol/L, multiply by 17.1.

‡ Normal range, 10.0 ng/mL.

The degree of activity and stage of fibrosis were assessed according to Ishak scoring system (13). The tumors were graded using the Edmondson-Steiner grading system (14).
