# Supplementary material for: Analysis of long noncoding RNA expression in hepatocellular carcinoma of different viral etiology
Source: J Transl Med. 2016 Nov 28;14:328. doi: 10.1186/s12967-016-1085-4 (PMC5125040; doi:10.1186/s12967-016-1085-4)
Supplement: Supplementary file 3 — Additional file 3: Table S3. Seventeen lncRNAs significantly dysregulated on profiling data in HCC patients. [file 12967_2016_1085_MOESM3_ESM.docx]

**Table S3. Seventeen lncRNAs significantly dysregulated on profiling data in HCC patients**

| **LncRNA** | ***P*-value** | | |  | **Fold change** | | |
| --- | --- | --- | --- | --- | --- | --- | --- |
|  | HBV  HCC VS NT | HCV  HCC VS NT | HDV  HCC VS NT |  | HBV  HCC VS NT | HCV  HCC VS NT | HDV  HCC VS NT |
| 7SK | **0.040** | **0.000** | 0.115 |  | +1.467 | +1.820 | +1.644 |
| aHIF | 0.646 | **0.010** | 0.801 |  | +1.112 | -1.433 | -1.104 |
| AK023948 | 0.568 | **0.025** | 0.241 |  | -1.247 | -3.364 | -2.247 |
| ANRIL | **0.002** | **0.000** | 0.070 |  | +5.129 | +4.852 | +6.644 |
| BC017743 | **0.015** | **0.004** | **0.001** |  | +8.385 | +6.895 | +10.940 |
| BC043430 | **0.019** | **0.006** | 0.503 |  | +8.072 | +7.486 | +1.690 |
| DLG2AS | **0.002** | 0.935 | 0.525 |  | -1.988 | +1.044 | -1.248 |
| HOTTIP | **0.016** | **0.022** | 0.083 |  | +23.349 | +44.959 | +16.405 |
| IPW | 0.502 | **0.030** | 0.656 |  | +1.454 | -2.216 | +1.162 |
| LINC01152 | **0.001** | **0.040** | **0.001** |  | -3.057 | -5.727 | -6.093 |
| MALAT1 | 0.053 | **0.017** | 0.200 |  | +1.487 | +1.469 | +1.363 |
| PAR5 | 0.997 | **0.002** | 0.997 |  | +1.002 | -2.481 | +1.001 |
| PCAT-29 | **0.017** | 0.373 | 0.386 |  | -1.999 | +1.873 | +2.078 |
| PTENP1 | **0.002** | 0.692 | 0.843 |  | -1.458 | +1.057 | -1.058 |
| ST7OT1 | **0.032** | 0.104 | 0.817 |  | +2.450 | +1.680 | +1.088 |
| TMEVPG1 | 0.405 | **0.033** | 0.073 |  | -1.501 | -4.761 | -2.713 |
| Y3 | 0.423 | 0.987 | **0.000** |  | -1.193 | +1.003 | -1.879 |

HCC denotes hepatocellular carcinoma; NT, surrounding nontumorous tissue; HBV, hepatitis B virus; HCV, hepatitis C virus; HDV, hepatitis D virus. *P*-values were calculated by Student’s paired *t*-test. The positive sign (+) indicates upregulation of lncRNA expression in HCC; the negative sign (-) indicates downregulation of lncRNA expression in HCC.
