# Supplementary material for: Analysis of long noncoding RNA expression in hepatocellular carcinoma of different viral etiology
Source: J Transl Med. 2016 Nov 28;14:328. doi: 10.1186/s12967-016-1085-4 (PMC5125040; doi:10.1186/s12967-016-1085-4)
Supplement: Supplementary file 4 — Additional file 4: Table S4. Relative expression levels of seventeen dysregulated lncRNAs in HCC patients. [file 12967_2016_1085_MOESM4_ESM.docx]

**Table S4. Relative expression levels of seventeen dysregulated lncRNAs in HCC patients**

| **LncRNA** | **Non-tumor** | | |  | **HCC** | | | | |
| --- | --- | --- | --- | --- | --- | --- | --- | --- | --- |
|  | HBV | HCV | HDV |  | HBV | | HCV | | HDV |
| 7SK | 1.856±1.352 | 1.253±0.513 | 0.761±0.173 |  | 2.438±2.437 | 1.908±0.994 | | 1.227±0.268 | |
| aHIF | 2.047±1.197 | 1.858±1.078 | 0.978±0.488 |  | 2.374±4.045 | 0.716±0.786 | | 0.361±0.137 | |
| AK023948 | 1.797±1.647 | 1.433±0.681 | 1.863±0.848 |  | 1.211±1.479 | 0.819±0.628 | | 0.616±0.668 | |
| ANRIL | 2.991±2.382 | 8.071±8.411 | 11.280±7.032 |  | 24.916±25.705 | 35.556±14.585 | | 34.463±25.289 | |
| BC017743 | 1.101±0.733 | 2.213±1.672 | 3.089±2.167 |  | 10.217±8.410 | 18.507±16.433 | | 24.016±12.239 | |
| BC043430 | 1.202±0.848 | 2.237±1.652 | 3.568±2.245 |  | 13.894±12.200 | 22.131±21.162 | | 30.512±12.634 | |
| DLG2AS | 1.095±0.552 | 0.985±0.529 | 0.718±0.305 |  | 0.643±0.482 | 1.615±2.788 | | 0.694±0.941 | |
| HOTTIP | 1.961±3.070 | 0.406±0.408 | 0.277±0.160 |  | 8.751±9.127 | 44.746±44.894 | | 19.470±18.018 | |
| IPW | 1.022±0.585 | 1.213±0.394 | 0.895±0.269 |  | 1.422±1.978 | 0.716±0.573 | | 0.782±0.409 | |
| LINC01152 | 1.490±1.083 | 2.687±1.620 | 2.380±1.144 |  | 0.574±0.919 | 0.603±0.606 | | 0.195±0.151 | |
| MALAT1 | 1.349±1.248 | 1.772±1.762 | 0.796±0.479 |  | 2.608±2.120 | 2.758±3.631 | | 1.031±0.671 | |
| PAR5 | 1.153±0.637 | 1.046±0.485 | 0.860±0.224 |  | 1.027±1.008 | 0.504±0.385 | | 0.567±0.318 | |
| PCAT-29 | 0.672±0.335 | 0.690±0.642 | 0.709±0.866 |  | 0.298±0.398 | 1.693±3.422 | | 0.352±0.278 | |
| PTENP1 | 5.831±6.936 | 1.701±1.153 | 1.574±0.269 |  | 5.103±8.775 | 0.979±0.900 | | 0.712±0.595 | |
| ST7OT1 | 0.525±0.686 | 0.589±0.676 | 0.339±0.321 |  | 0.916±1.684 | 1.229±1.041 | | 0.639±0.366 | |
| TMEVPG1 | 2.492±3.277 | 3.770±2.387 | 6.569±1.708 |  | 0.577±0.687 | 0.695±0.646 | | 1.286±1.428 | |
| Y3 | 1.816±0.931 | 1.402±0.630 | 0.978±0.231 |  | 1.280±1.001 | 1.510±1.142 | | 0.521±0.158 | |

| **LncRNA** | **Liver cirrhosis** | | |
| --- | --- | --- | --- |
|  | HBV | HCV | HDV |
| 7SK | 1.966±0.927 | 1.110±0.323 | 0.703±0.107 |
| aHIF | 1.224±0.404 | 1.617±1.535 | 1.475±1.079 |
| AK023948 | 1.196±0.625 | 2.725±0.926 | 1.016±0.573 |
| ANRIL | 3.859±2.118 | 13.010±8.300 | 4.007±2.735 |
| BC017743 | 0.781±0.409 | 1.296±0.507 | 0.418±0.138 |
| BC043430 | 0.784±0.222 | 1.837±0.794 | 0.496±0.253 |
| DLG2AS | 0.413±0.196 | 0.523±0.179 | 0.161±0.072 |
| HOTTIP | 0.760±0.854 | 0.797±0.433 | 0.266±0.168 |
| IPW | 0.847±0.037 | 1.502±0.424 | 0.720±0.146 |
| LINC01152 | 0.180±0.111 | 2.028±1.361 | 1.282±1.006 |
| MALAT1 | 0.382±0.020 | 0.526±0.152 | 0.408±0.110 |
| PAR5 | 1.207±0.247 | 1.722±0.464 | 0.598±0.131 |
| PCAT-29 | 1.198±0.838 | 0.347±0.170 | 0.375±0.297 |
| PTENP1 | 1.851±2.008 | 1.485±1.091 | 1.748±1.012 |
| ST7OT1 | 0.143±0.084 | 0.188±0.144 | 1.039±0.428 |
| TMEVPG1 | 1.411±0.203 | 8.294±4.900 | 4.002±3.455 |
| Y3 | 1.119±0.226 | 1.278±0.372 | 0.793±0.166 |

| **LncRNA** | **Normal liver** | |
| --- | --- | --- |
|  | Liver donor | Angioma |
| 7SK | 0.498±0.173 | 1.627±0.372 |
| aHIF | 0.898±0.935 | 1.127±0.682 |
| AK023948 | 0.831±0.525 | 1.211±0.676 |
| ANRIL | 0.519±0.245 | 1.602±1.941 |
| BC017743 | 1.241±1.205 | 0.690±0.177 |
| BC043430 | 1.190±0.996 | 0.770±0.223 |
| DLG2AS | 1.299±2.392 | 0.626±0.255 |
| HOTTIP | 1.087±0.652 | 0.877±0.365 |
| IPW | 0.805±0.416 | 1.244±0.313 |
| LINC01152 | 1.459±1.378 | 0.426±0.264 |
| MALAT1 | 1.252±0.798 | 0.685±0.075 |
| PAR5 | 0.701±0.369 | 1.374±0.426 |
| PCAT-29 | 0.355±0.345 | 1.806±0.676 |
| PTENP1 | 0.757±0.729 | 1.304±0.697 |
| ST7OT1 | 1.525±0.802 | 0.343±0.252 |
| TMEVPG1 | 1.114±1.048 | 0.857±0.405 |
| Y3 | 0.401±0.124 | 1.749±0.463 |

HCC denotes hepatocellular carcinoma; HBV, hepatitis B virus; HCV, hepatitis C virus; HDV, hepatitis D virus. Data are expressed as mean ± standard deviation.
