# Supplementary material for: Analysis of long noncoding RNA expression in hepatocellular carcinoma of different viral etiology
Source: J Transl Med. 2016 Nov 28;14:328. doi: 10.1186/s12967-016-1085-4 (PMC5125040; doi:10.1186/s12967-016-1085-4)
Supplement: Supplementary file 5 — Additional file 5: Table S5. Eighteen lncRNAs previously reported to be associated with HCC. [file 12967_2016_1085_MOESM5_ESM.docx]

**Table S5. Fourteen lncRNAs significantly dysregulated in HCC patients**

| **LncRNA** | ***P*-value** | | |  | **Fold change** | | |
| --- | --- | --- | --- | --- | --- | --- | --- |
|  | HBV  HCC VS NT | HCV  HCC VS NT | HDV  HCC VS NT |  | HBV  HCC VS NT | HCV  HCC VS NT | HDV  HCC VS NT |
| 7SK | 0.378 | **0.009** | **0.021** |  | +1.313 | +1.514 | +1.612 |
| aHIF | 0.760 | **0.001** | 0.066 |  | +1.159 | **-2.594** | -2.707 |
| ANRIL | **0.018** | **0.001** | 0.150 |  | **+8.331** | **+4.406** | +3.055 |
| BC017743 | **0.005** | **0.010** | **0.031** |  | **+9.279** | **+8.362** | **+7.774** |
| BC043430 | **0.006** | **0.014** | **0.024** |  | **+11.562** | **+9.895** | **+8.551** |
| DLG2AS | **0.021** | 0.443 | 0.966 |  | -1.702 | +1.640 | -1.035 |
| HOTTIP | **0.039** | **0.012** | 0.124 |  | **+4.464** | **+110.293** | +70.210 |
| IPW | 0.419 | **0.038** | 0.492 |  | +1.392 | -1.695 | -1.144 |
| LINC01152 | **0.038** | **0.003** | **0.041** |  | **-2.596** | **-4.453** | **-12.191** |
| MALAT1 | **0.013** | 0.256 | 0.138 |  | +1.933 | +1.557 | +1.295 |
| PAR5 | 0.580 | **0.005** | 0.121 |  | -1.123 | **-2.074** | -1.518 |
| PCAT-29 | **0.004** | 0.343 | 0.515 |  | **-2.256** | +2.454 | -2.012 |
| TMEVPG1 | 0.099 | **0.005** | **0.036** |  | -4.318 | **-5.424** | **-5.108** |
| Y3 | 0.198 | 0.653 | **0.045** |  | -1.419 | +1.077 | **-2.052** |

HCC denotes hepatocellular carcinoma; NT, surrounding nontumorous tissue; HBV, hepatitis B virus; HCV, hepatitis C virus; HDV, hepatitis D virus. *P*-values were calculated by Student’s paired *t*-test. The positive sign (+) indicates upregulation of lncRNA expression in HCC; the negative sign (- ) indicates downregulation of lncRNA expression in HCC.
