# Supplementary material for: Analysis of long noncoding RNA expression in hepatocellular carcinoma of different viral etiology
Source: J Transl Med. 2016 Nov 28;14:328. doi: 10.1186/s12967-016-1085-4 (PMC5125040; doi:10.1186/s12967-016-1085-4)
Supplement: Supplementary file 6 — Additional file 6: Table S6. Relative expression levels of eighteen previously identified HCC-associated lncRNAs. [file 12967_2016_1085_MOESM6_ESM.docx]

**Table S6. Relative expression levels of eighteen previously identified HCC-associated lncRNAs**

| **LncRNA** | **Non-tumor** | | |  | **HCC** | | | | |
| --- | --- | --- | --- | --- | --- | --- | --- | --- | --- |
|  | HBV | HCV | HDV |  | HBV | | HCV | | HDV |
| AFAP1-AS1 | 1.416±0.721 | 2.349±2.018 | 2.265±0.810 |  | 18.434±34.553 | 3.322±2.368 | | 11.754±18.210 | |
| CCAT1 | 3.611±4.122 | 1.165±1.146 | 0.988±0.514 |  | 2.904±3.986 | 0.556±0.441 | | 1.933±3.036 | |
| DANCR | 0.605±0.197 | 0.521±0.315 | 0.601±0.292 |  | 0.922±0.652 | 1.050±1.385 | | 1.040±0.454 | |
| DBH-AS1 | 0.830±0.352 | 0.615±0.339 | 0.636±0.411 |  | 0.268±0.271 | 0.581±0.504 | | 0.388±0.154 | |
| hDREH | 1.105±0.837 | 0.316±0.157 | 0.483±0.308 |  | 0.647±0.423 | 0.829±0.544 | | 0.637±0.369 | |
| GAS5 | 1.109±0.382 | 1.411±1.000 | 1.122±0.255 |  | 1.647±1.191 | 1.867±1.039 | | 1.251±0.660 | |
| HEIH | 1.614±1.855 | 2.238±2.213 | 1.525±1.316 |  | 3.083±3.452 | 3.318±3.576 | | 1.606±0.907 | |
| LET | 0.904±0.265 | 0.830±0.212 | 0.620±0.121 |  | 0.904±0.786 | 1.002±0.393 | | 0.398±0.150 | |
| Linc00152 | 3.572±4.675 | 4.556±3.339 | 3.551±0.903 |  | 5.598±3.644 | 5.199±3.101 | | 4.719±2.324 | |
| LincTCF7 | 0.698±0.787 | 0.369±0.209 | 0.238±0.123 |  | 1.089±1.088 | 0.801±0.787 | | 0.324±0.297 | |
| MVIH | 1.306±1.120 | 2.134±1.007 | 1.892±0.952 |  | 1.241±0.884 | 2.277±1.078 | | 1.559±0.555 | |
| PCNA-AS1 | 1.107±0.320 | 1.114±0.462 | 1.202±0.318 |  | 1.978±1.557 | 2.299±0.750 | | 1.796±0.684 | |
| hPVT1 | 2.143±2.174 | 3.678±2.792 | 3.035±1.969 |  | 4.043±2.795 | 3.196±2.988 | | 2.725±2.069 | |
| uc.338 | 1.588±1.194 | 1.960±1.489 | 1.376±1.019 |  | 1.882±1.551 | 2.132±2.308 | | 1.072±0.592 | |
| UCA1 | 1.056±0.635 | 1.925±2.188 | 1.129±0.888 |  | 0.637±0.757 | 1.278±2.160 | | 0.490±0.530 | |
| UFC1 | 0.674±0.289 | 0.658±0.459 | 0.645±0.330 |  | 1.014±0.694 | 1.543±0.884 | | 0.907±0.630 | |
| ZEB1-AS1 | 0.769±0.246 | 0.961±0.288 | 0.922±0.300 |  | 1.212±0.544 | 1.577±0.863 | | 1.321±1.072 | |
| ZFAS1 | 1.028±0.386 | 1.340±0.774 | 1.197±0.344 |  | 1.218±0.681 | 1.367±0.813 | | 1.097±0.722 | |

| **LncRNA** | **Liver cirrhosis** | | |
| --- | --- | --- | --- |
|  | HBV | HCV | HDV |
| AFAP1-AS1 | 2.967±2.902 | 4.063±2.137 | 1.871±1.406 |
| CCAT1 | 1.163±0.603 | 0.746±0.351 | 0.576±0.434 |
| DANCR | 0.729±0.446 | 0.474±0.191 | 0.758±0.360 |
| DBH-AS1 | 0.601±0.449 | 0.434±0.153 | 0.695±0.323 |
| hDREH | 0.874±0.610 | 0.891±0.567 | 0.390±0.125 |
| GAS5 | 1.148±0.412 | 1.685±0.688 | 1.080±0.388 |
| HEIH | 0.374±0.161 | 0.876±0.262 | 0.633±0.235 |
| LET | 0.789±0.333 | 1.645±0.962 | 0.840±0.388 |
| Linc00152 | 3.780±3.072 | 4.205±2.464 | 4.693±2.906 |
| LincTCF7 | 1.186±1.382 | 0.489±0.320 | 0.259±0.137 |
| MVIH | 0.436±0.164 | 0.711±0.261 | 0.476±0.181 |
| PCNA-AS1 | 0.941±0.077 | 0.982±0.349 | 1.071±0.360 |
| hPVT1 | 0.453±0.181 | 1.110±0.566 | 0.619±0.368 |
| uc.338 | 0.354±0.032 | 0.589±0.181 | 0.437±0.145 |
| UCA1 | 1.614±1.313 | 3.774±5.416 | 0.904±0.530 |
| UFC1 | 0.879±0.488 | 0.892±0.412 | 0.863±0.477 |
| ZEB1-AS1 | 0.790±0.081 | 0.806±0.265 | 1.265±0.093 |
| ZFAS1 | 1.137±0.272 | 1.517±0.519 | 1.129±0.346 |

| **LncRNA** | **Normal liver** | |
| --- | --- | --- |
|  | Liver donor | Angioma |
| AFAP1-AS1 | 0.989±0.286 | 1.013±0.493 |
| CCAT1 | 1.422±1.457 | 0.472±0.244 |
| DANCR | 1.028±0.436 | 0.965±0.403 |
| DBH-AS1 | 0.597±0.435 | 1.503±0.302 |
| hDREH | 1.174±0.833 | 0.778±0.310 |
| GAS5 | 0.841±0.285 | 1.199±0.222 |
| HEIH | 1.320±1.014 | 0.600±0.084 |
| LET | 1.203±0.792 | 0.747±0.236 |
| Linc00152 | 1.187±0.805 | 0.766±0.266 |
| LincTCF7 | 1.167±1.353 | 0.791±0.353 |
| MVIH | 1.232±0.856 | 0.662±0.142 |
| PCNA-AS1 | 0.797±0.402 | 1.253±0.206 |
| hPVT1 | 1.396±1.181 | 0.510±0.073 |
| uc.338 | 1.362±1.058 | 0.548±0.066 |
| UCA1 | 1.071±1.122 | 0.911±0.630 |
| UFC1 | 0.889±0.550 | 1.138±0.399 |
| ZEB1-AS1 | 1.005±0.558 | 0.994±0.222 |
| ZFAS1 | 0.833±0.307 | 1.209±0.308 |

HCC denotes hepatocellular carcinoma; HBV, hepatitis B virus; HCV, hepatitis C virus; HDV, hepatitis D virus. Data are expressed as mean ± standard deviation.
