# Supplementary material for: Analysis of long noncoding RNA expression in hepatocellular carcinoma of different viral etiology
Source: J Transl Med. 2016 Nov 28;14:328. doi: 10.1186/s12967-016-1085-4 (PMC5125040; doi:10.1186/s12967-016-1085-4)
Supplement: Supplementary file 7 — Additional file 7: Table S7. Eighteen lncRNAs previously reported to be associated with HCC. [file 12967_2016_1085_MOESM7_ESM.docx]

**Table S7. Eighteen lncRNAs previously reported to be associated with HCC**

| **LncRNA** | ***P*-value** | | |  | **Fold change** | | |
| --- | --- | --- | --- | --- | --- | --- | --- |
|  | HBV  HCC VS NT | HCV  HCC VS NT | HDV  HCC VS NT |  | HBV  HCC VS NT | HCV  HCC VS NT | HDV  HCC VS NT |
| AFAP1-AS1 | 0.133 | 0.111 | 0.372 |  | +13.019 | +1.414 | +5.190 |
| CCAT1 | 0.599 | 0.173 | 0.530 |  | -1.243 | -2.094 | +1.956 |
| DANCR | 0.159 | 0.242 | 0.250 |  | +1.524 | +2.017 | +1.729 |
| DBH-AS1 | **0.000** | 0.805 | 0.333 |  | -3.091 | -1.058 | -1.636 |
| hDREH | 0.052 | **0.007** | 0.615 |  | -1.709 | +2.622 | +1.319 |
| GAS5 | 0.187 | 0.247 | 0.728 |  | +1.485 | +1.323 | +1.115 |
| HEIH | **0.025** | 0.096 | 0.800 |  | +1.910 | +1.482 | +1.053 |
| LET | 1.000 | 0.203 | 0.174 |  | +1.000 | +1.208 | -1.560 |
| Linc00152 | 0.309 | 0.669 | 0.397 |  | +1.567 | +1.141 | +1.329 |
| LincTCF7 | 0.126 | 0.125 | 0.419 |  | +1.559 | +2.168 | +1.359 |
| MVIH | 0.595 | 0.642 | 0.208 |  | -1.053 | +1.067 | -1.214 |
| PCNA-AS1 | 0.074 | **0.000** | 0.106 |  | +1.787 | +2.064 | +1.494 |
| hPVT1 | **0.004** | 0.474 | 0.109 |  | +1.887 | -1.151 | -1.114 |
| uc.338 | 0.278 | 0.754 | 0.262 |  | +1.186 | +1.088 | -1.284 |
| UCA1 | 0.184 | 0.202 | 0.358 |  | -1.658 | -1.507 | -2.305 |
| UFC1 | 0.153 | **0.030** | 0.368 |  | +1.504 | +2.346 | +1.406 |
| ZEB1-AS1 | **0.028** | **0.036** | 0.458 |  | +1.574 | +1.642 | +1.432 |
| ZFAS1 | 0.437 | 0.893 | 0.744 |  | +1.186 | +1.020 | -1.091 |

HCC denotes hepatocellular carcinoma; NT, surrounding nontumorous tissue; HBV, hepatitis B virus; HCV, hepatitis C virus; HDV, hepatitis D virus. *P*-values were calculated by Student’s paired *t*-test. The positive sign (+) indicates upregulation of lncRNA expression in HCC; the negative sign (-) indicates downregulation of lncRNA expression in HCC.
