# Supplementary material for: Analysis of long noncoding RNA expression in hepatocellular carcinoma of different viral etiology
Source: J Transl Med. 2016 Nov 28;14:328. doi: 10.1186/s12967-016-1085-4 (PMC5125040; doi:10.1186/s12967-016-1085-4)
Supplement: Supplementary file 8 — Additional file 8: Figure S1. Expression of HCC-associated lncRNAs in the tumor and surrounding non-tumorous tissue in a series of patients with HCC associated with HBV, HCV and HDV analyzed by qRT-PCR. [file 12967_2016_1085_MOESM8_ESM.pptx]

## Slide 1
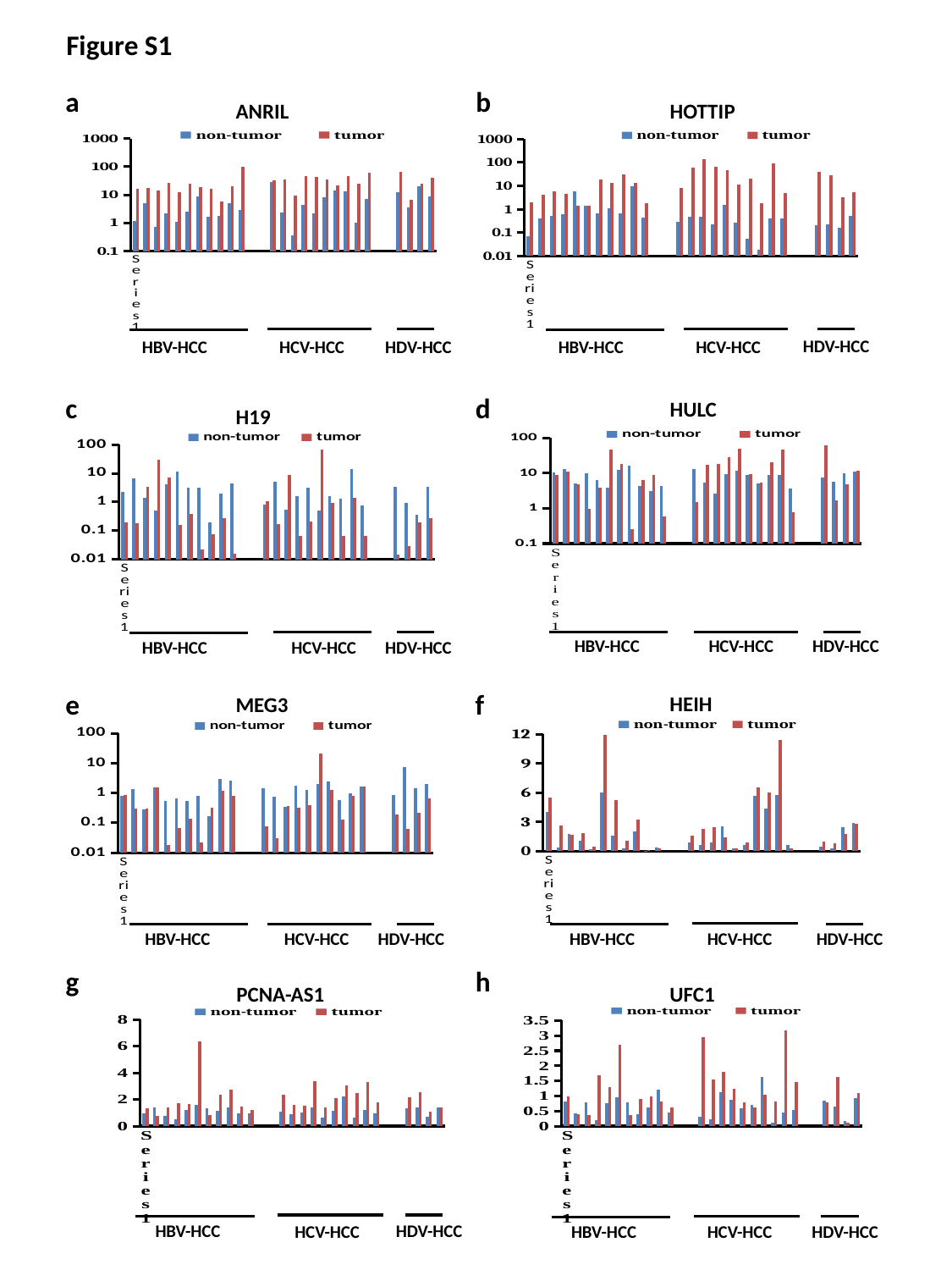

Figure S1
a
b
HOTTIP
ANRIL
### Chart
| Category | non-tumor | tumor |
|---|---|---|
| | 0.0672250459884271 | 1.96262786332602 |
| | 0.38712480257394 | 4.271277547368758 |
| | 0.5312966963945 | 5.863244208316686 |
| | 0.624330411555189 | 4.448744201318708 |
| | 5.98841683864185 | 1.399655858779174 |
| | 1.355485938688408 | 1.390596773499858 |
| | 0.676337919554943 | 18.2072565176761 |
| | 1.054284072263325 | 13.13557679936098 |
| | 0.640507401573357 | 29.9082615021544 |
| | 9.790293749101398 | 13.9183245349513 |
| | 0.451573943289911 | 1.75966420527228 |
| | None | None |
| | None | None |
| | 0.285042045610557 | 7.896676817497441 |
| | 0.456725468759332 | 58.69619781498665 |
| | 0.493315713559603 | 138.0262778309662 |
| | 0.228839205739608 | 64.37212311150576 |
| | 1.478307248182626 | 46.9953186427088 |
| | 0.269429391896737 | 11.27106664491657 |
| | 0.0531878911667694 | 20.2925905672849 |
| | 0.0185466977499471 | 1.757038918255191 |
| | 0.386256642160162 | 93.18660248832033 |
| | 0.387360471939522 | 4.964488724597661 |
| | None | None |
| | None | None |
| | 0.21031499899586 | 40.017736088087 |
| | 0.231207827708945 | 29.1343990418767 |
| | 0.155645486593376 | 3.364746105801957 |
| | 0.512106809279653 | 5.364955287180203 |
### Chart
| Category | non-tumor | tumor |
|---|---|---|
| | 1.168601657603381 | 15.80256387001324 |
| | 5.086744885392588 | 17.7877698811482 |
| | 0.733802762427696 | 14.40773095182704 |
| | 2.240292706794124 | 27.00272763232369 |
| | 1.128043138979788 | 12.46523987745583 |
| | 2.604523114645004 | 25.4569511603729 |
| | 8.729074745327818 | 18.22427150913072 |
| | 1.617595333914569 | 16.44921396833388 |
| | 1.792515061067417 | 5.636920578676766 |
| | 4.859989647866222 | 20.45549778828088 |
| | 2.935787896566405 | 100.3846409538108 |
| | None | None |
| | None | None |
| | 27.64631405395282 | 32.758644138657 |
| | 2.336896340462147 | 35.12884507007568 |
| | 0.353533232352423 | 9.5804056332164 |
| | 4.335140468360533 | 46.67558634520206 |
| | 2.177360424785892 | 43.81317135506022 |
| | 8.4192413590273 | 35.39762695845693 |
| | 14.15011370344627 | 21.29316852847909 |
| | 13.08962844123631 | 45.2255530704626 |
| | 1.057704951002501 | 24.99939438754982 |
| | 7.139666769680687 | 60.68843618754472 |
| | None | None |
| | None | None |
| | 12.53328976343417 | 66.33349982106918 |
| | 3.452182514420655 | 6.446126457078908 |
| | 20.21944848133446 | 25.10148377481176 |
| | 8.913587764733663 | 39.97112409848624 |HDV-HCC
HBV-HCC
HCV-HCC
HDV-HCC
HBV-HCC
HCV-HCC
c
d
HULC
H19
### Chart
| Category | non-tumor | tumor |
|---|---|---|
| | 10.44655643354622 | 8.719337670311178 |
| | 12.76811415828108 | 10.8278135903436 |
| | 5.016530947996944 | 4.705550622618769 |
| | 10.01733297955462 | 0.947209398226269 |
| | 6.205126193006022 | 3.786865157150775 |
| | 3.79265344418232 | 45.60890253394004 |
| | 12.17473993941088 | 18.11050726229714 |
| | 16.27723279610337 | 0.253273501699342 |
| | 4.25174345957415 | 6.54156601612615 |
| | 3.074982181617797 | 8.79779844920492 |
| | 4.364689575898335 | 0.587810506333283 |
| | None | None |
| | None | None |
| | 13.2028140233184 | 1.493483760607968 |
| | 5.27666374414294 | 17.02463013610544 |
| | 2.626370682341631 | 18.61893373997385 |
| | 9.218091260169299 | 28.54252476182172 |
| | 11.66474650731972 | 48.25598735600983 |
| | 8.836861362933863 | 9.336284670472317 |
| | 5.049795800736645 | 5.2832422059633 |
| | 8.8577424557244 | 20.30532270558162 |
| | 9.05859968954503 | 46.3867238172834 |
| | 3.629971070654348 | 0.778504913845413 |
| | None | None |
| | None | None |
| | 7.598022849358848 | 61.9238407527513 |
| | 5.664978774562894 | 1.666463594339772 |
| | 9.85304434742011 | 4.95977973152348 |
| | 10.84476247993797 | 11.71028311470024 |
### Chart
| Category | non-tumor | tumor |
|---|---|---|
| | 2.212940958266438 | 0.195008235854359 |
| | 6.778666340683256 | 0.187330856521765 |
| | 1.373077407745641 | 3.276414672634134 |
| | 0.499465855259051 | 30.48237610254311 |
| | 4.29522277637521 | 7.313989016873256 |
| | 11.23398175804908 | 0.159413637611481 |
| | 3.181260383833342 | 0.388139713890043 |
| | 3.257657565225762 | 0.0213997604930487 |
| | 0.195746030972367 | 0.0750050976335314 |
| | 1.989093316170238 | 0.273838169781738 |
| | 4.515885666252119 | 0.0158026624969388 |
| | None | None |
| | None | None |
| | 0.794378488184775 | 1.092972771259608 |
| | 4.994763817218089 | 0.169838384688525 |
| | 0.551590670325093 | 8.934936401389406 |
| | 1.658883371122334 | 0.0672734688333533 |
| | 3.077316774509267 | 0.202001856918627 |
| | 0.494863472177577 | 68.90032869059598 |
| | 1.643821286601502 | 0.952328332830022 |
| | 1.306180214581586 | 0.0667233003089602 |
| | 14.24636753597293 | 1.413741607873348 |
| | 0.751811537290226 | 0.0654677841647344 |
| | None | None |
| | None | None |
| | 3.445427405012615 | 0.0146839046355872 |
| | 0.925506136063829 | 0.0286735473753567 |
| | 0.35784902655167 | 0.200608474877571 |
| | 3.385363993239033 | 0.267148525640936 |HBV-HCC
HCV-HCC
HDV-HCC
HBV-HCC
HCV-HCC
HDV-HCC
e
f
HEIH
MEG3
### Chart
| Category | non-tumor | tumor |
|---|---|---|
| | 3.98077936498867 | 5.452233738594443 |
| | 0.41001395322025 | 2.630758673214546 |
| | 1.741571751517066 | 1.670308875395027 |
| | 1.050645822487756 | 1.819495410540225 |
| | 0.184256882257541 | 0.497386036778445 |
| | 5.979315811279705 | 11.90698637462414 |
| | 1.631354039225661 | 5.23500498937049 |
| | 0.290556354527444 | 1.062109795384598 |
| | 2.057383745665544 | 3.211760715099644 |
| | 0.0382250556498902 | 0.11138897975765 |
| | 0.38741679745016 | 0.315858305201311 |
| | None | None |
| | None | None |
| | 0.900058645657676 | 1.558826013302764 |
| | 0.633560111428091 | 2.305307037699034 |
| | 0.893463632482772 | 2.432461401121118 |
| | 2.555265876771521 | 1.402969759139471 |
| | 0.320488481583922 | 0.309825525317546 |
| | 0.594693170191713 | 0.934571153125705 |
| | 5.687067438384633 | 6.531359919688886 |
| | 4.34041927973694 | 6.030664229270122 |
| | 5.791027758309663 | 11.37827864687856 |
| | 0.666865820439054 | 0.293309847197394 |
| | None | None |
| | None | None |
| | 0.466336226440938 | 0.986972923875228 |
| | 0.324255884663561 | 0.847365567926163 |
| | 2.453492586514745 | 1.764751334393078 |
| | 2.85688291764139 | 2.824266858817245 |
### Chart
| Category | non-tumor | tumor |
|---|---|---|
| | 0.806743590256491 | 0.844196419418008 |
| | 1.331602159598352 | 0.293026615538383 |
| | 0.281792792183569 | 0.303376788860512 |
| | 1.490238371007593 | 1.49101690553365 |
| | 0.528961496108122 | 0.0178878104428892 |
| | 0.652307271231576 | 0.0658981655766079 |
| | 0.532141607412028 | 0.134256246187214 |
| | 0.796566722251156 | 0.0218228239741114 |
| | 0.164195286216665 | 0.315425590374095 |
| | 2.944337628503604 | 1.20077314371362 |
| | 2.655870155085411 | 0.824927430671685 |
| | None | None |
| | None | None |
| | 1.459494845177212 | 0.0776115735453043 |
| | 0.737315308004609 | 0.0300478195709685 |
| | 0.34979404055307 | 0.37132506697827 |
| | 1.739758571518868 | 0.317545254528064 |
| | 1.253755574447678 | 0.394483979094113 |
| | 2.027753965009357 | 21.35017685441193 |
| | 2.382640211977646 | 1.228360150467151 |
| | 0.560895578440655 | 0.128388698178215 |
| | 0.976192867129177 | 0.792393041047325 |
| | 1.597952047369363 | 1.592292208405182 |
| | None | None |
| | None | None |
| | 0.857242400857058 | 0.1959244178369 |
| | 7.361244116642712 | 0.0615476407026532 |
| | 1.477842594108431 | 0.21667252276259 |
| | 2.031265209258732 | 0.676610333244856 |HBV-HCC
HCV-HCC
HDV-HCC
HBV-HCC
HCV-HCC
HDV-HCC
g
h
PCNA-AS1
UFC1
### Chart
| Category | non-tumor | tumor |
|---|---|---|
| | 0.95942979791215 | 1.335724664068802 |
| | 1.422493097954981 | 0.76331537310267 |
| | 0.748834678375182 | 1.430057496213584 |
| | 0.496155637946028 | 1.75117425811734 |
| | 1.233845413162695 | 1.62476059129626 |
| | 1.563029115425284 | 6.333470036242125 |
| | 1.32767280809347 | 0.805191391074187 |
| | 1.136892348119381 | 2.323454112714583 |
| | 1.386918153867597 | 2.723705371154573 |
| | 0.944085320615708 | 1.462481781181808 |
| | 0.957252980709969 | 1.20634758628872 |
| | None | None |
| | None | None |
| | 1.07190730558715 | 2.384654327399537 |
| | 0.896929285982446 | 1.622374242144303 |
| | 0.998320635493241 | 1.522936883901596 |
| | 1.399889208575961 | 3.364129290608898 |
| | 0.612847553838742 | 1.39308438670875 |
| | 1.152297031009788 | 2.078780819161036 |
| | 2.235364585363723 | 3.060981132881157 |
| | 0.626871068351526 | 2.469342840677876 |
| | 1.194017227715492 | 3.339044110980777 |
| | 0.949861881216913 | 1.752882330531088 |
| | None | None |
| | None | None |
| | 1.33275551541371 | 2.146158537577433 |
| | 1.378303070615254 | 2.573096657579781 |
| | 0.725086064789409 | 1.087200219493934 |
| | 1.370479409163311 | 1.375646847356753 |
### Chart
| Category | non-tumor | tumor |
|---|---|---|
| | 0.817645922989523 | 0.982939231588037 |
| | 0.410683141870268 | 0.394699920146416 |
| | 0.796704502034277 | 0.365078757727688 |
| | 0.195783980804112 | 1.684694072043671 |
| | 0.764923105668638 | 1.297735737764557 |
| | 0.94339027403494 | 2.71152906070978 |
| | 0.790321813019827 | 0.379077736195887 |
| | 0.395574581984341 | 0.903934807047961 |
| | 0.629972227040793 | 0.987065092698133 |
| | 1.208993620945096 | 0.820429420350577 |
| | 0.464260399581336 | 0.62909710684182 |
| | None | None |
| | None | None |
| | 0.317288498917517 | 2.953676107304557 |
| | 0.229525067766382 | 1.54333754786235 |
| | 1.13297272664382 | 1.806377138334004 |
| | 0.881955415907173 | 1.232653772215828 |
| | 0.578717482561354 | 0.782065112589439 |
| | 0.70848446890963 | 0.62357052118467 |
| | 1.637559278862093 | 1.028358027265997 |
| | 0.123104988755358 | 0.825717468967801 |
| | 0.447572137396389 | 3.174774521228461 |
| | 0.521960534059609 | 1.464375084629847 |
| | None | None |
| | None | None |
| | 0.83690230870734 | 0.789145173618042 |
| | 0.644132831180086 | 1.631813445008228 |
| | 0.180202650031687 | 0.119498228185478 |
| | 0.917005716450556 | 1.085797925396204 |HBV-HCC
HDV-HCC
HCV-HCC
HBV-HCC
HCV-HCC
HDV-HCC

## Slide 2
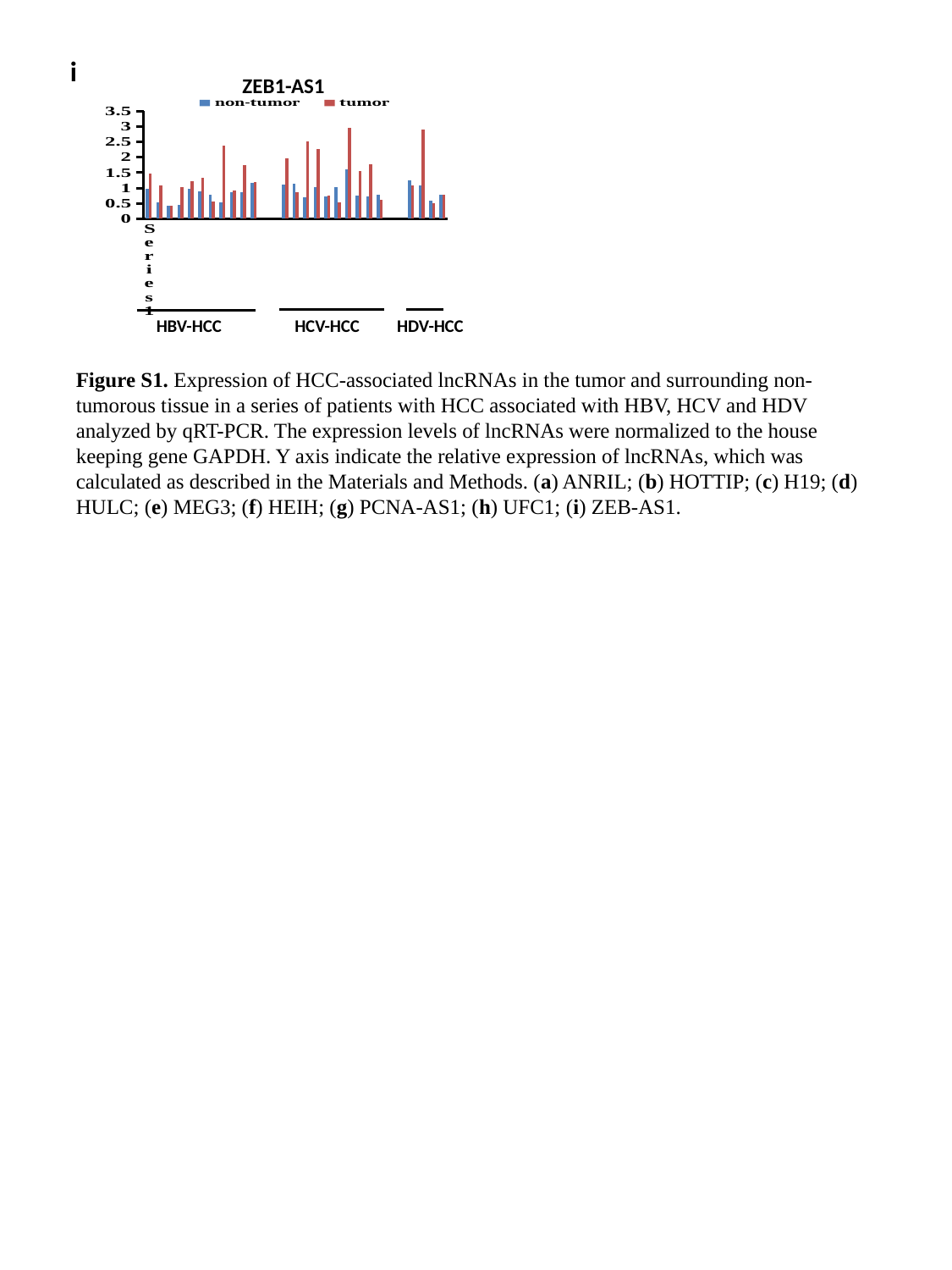

i
ZEB1-AS1
### Chart
| Category | non-tumor | tumor |
|---|---|---|
| | 0.975492488820064 | 1.471722499088232 |
| | 0.536126431988794 | 1.091039375564751 |
| | 0.420757872280418 | 0.414278590233882 |
| | 0.458735406906395 | 1.036619666388088 |
| | 0.969979168712223 | 1.216641962547336 |
| | 0.890676644738073 | 1.330008687582287 |
| | 0.784451778028962 | 0.548589026590937 |
| | 0.527649079711126 | 2.384422958294844 |
| | 0.869806363921604 | 0.908954617053397 |
| | 0.862455347041112 | 1.745013465843588 |
| | 1.168706501160214 | 1.180262014864178 |
| | None | None |
| | None | None |
| | 1.113696145284244 | 1.951823596062556 |
| | 1.13057889378704 | 0.850504143405761 |
| | 0.694273335706716 | 2.517858432703346 |
| | 1.041447900875444 | 2.27279122947698 |
| | 0.730704840444448 | 0.740536201577315 |
| | 1.02424877645238 | 0.533940418971576 |
| | 1.614589785043733 | 2.967113612291717 |
| | 0.750627821803588 | 1.544429663755319 |
| | 0.724700185844031 | 1.765974693691957 |
| | 0.78087101605657 | 0.625109645796379 |
| | None | None |
| | None | None |
| | 1.246759476993976 | 1.094135313340232 |
| | 1.086688288563703 | 2.887429395021351 |
| | 0.582304902189993 | 0.506901265012118 |
| | 0.772679308287513 | 0.793959966212263 |HBV-HCC
HCV-HCC
HDV-HCC
Figure S1. Expression of HCC-associated lncRNAs in the tumor and surrounding non-tumorous tissue in a series of patients with HCC associated with HBV, HCV and HDV analyzed by qRT-PCR. The expression levels of lncRNAs were normalized to the house keeping gene GAPDH. Y axis indicate the relative expression of lncRNAs, which was calculated as described in the Materials and Methods. (a) ANRIL; (b) HOTTIP; (c) H19; (d) HULC; (e) MEG3; (f) HEIH; (g) PCNA-AS1; (h) UFC1; (i) ZEB-AS1.
