# Supplementary material for: Analysis of long noncoding RNA expression in hepatocellular carcinoma of different viral etiology
Source: J Transl Med. 2016 Nov 28;14:328. doi: 10.1186/s12967-016-1085-4 (PMC5125040; doi:10.1186/s12967-016-1085-4)
Supplement: Supplementary file 9 — Additional file 9: Figure S2. The lncRNAs, DBH-AS1, hDREH and hPVT1 were differentially dysregulated in HBV-, HCV-, and HDV-related HCC. [file 12967_2016_1085_MOESM9_ESM.pptx]

## Slide 1
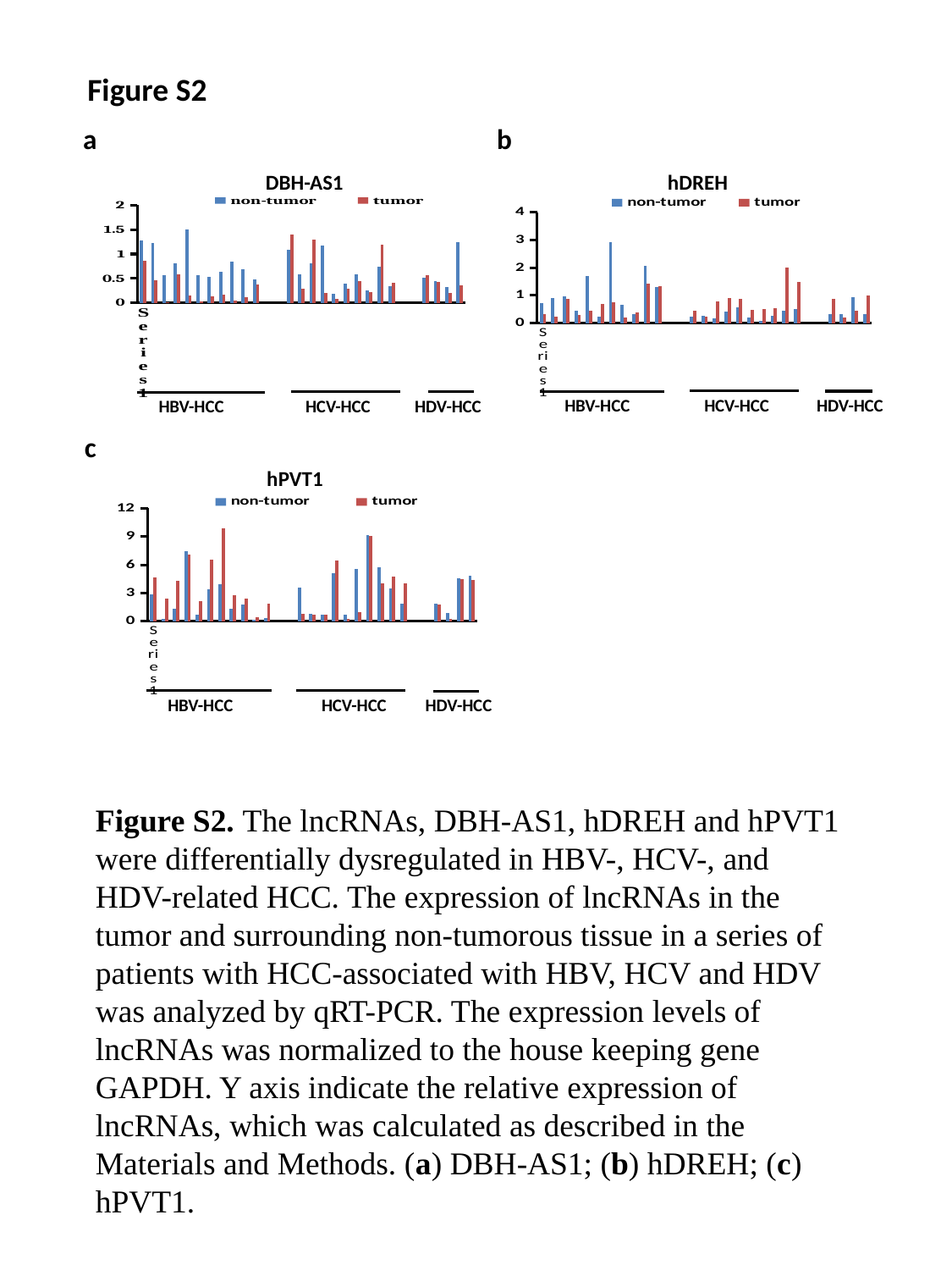

Figure S2
a
b
DBH-AS1
hDREH
### Chart
| Category | non-tumor | tumor |
|---|---|---|
| | 1.273973702323455 | 0.865451571543175 |
| | 1.232320481823538 | 0.463833362104653 |
| | 0.563752564949087 | 0.0194389822530934 |
| | 0.81430772708041 | 0.579110876276396 |
| | 1.510179456979018 | 0.149919089092029 |
| | 0.563771712685801 | 0.0266397803508573 |
| | 0.539094463185327 | 0.137614175885605 |
| | 0.631503877494088 | 0.174567693752383 |
| | 0.840759743030687 | 0.040791333966616 |
| | 0.684342284595883 | 0.122905893144108 |
| | 0.473665827341239 | 0.372774607981894 |
| | None | None |
| | None | None |
| | 1.084760083875476 | 1.399155196549711 |
| | 0.5887985392938 | 0.285499870723547 |
| | 0.812006678022859 | 1.286100601242363 |
| | 1.175903358613644 | 0.205692200782119 |
| | 0.176812968254939 | 0.076273171382845 |
| | 0.399546410198398 | 0.290453978697946 |
| | 0.580643674712543 | 0.440340973431814 |
| | 0.254116242567682 | 0.228837040624264 |
| | 0.739781176663663 | 1.196252547837926 |
| | 0.34006866844841 | 0.404110991529097 |
| | None | None |
| | None | None |
| | 0.520314925489651 | 0.571428935626249 |
| | 0.452093787342037 | 0.423597945564357 |
| | 0.329121914768711 | 0.198890944572988 |
| | 1.240706176444808 | 0.35961011281414 |
### Chart
| Category | non-tumor | tumor |
|---|---|---|
| | 0.730736267686496 | 0.314497884461289 |
| | 0.91450864782901 | 0.242003399313466 |
| | 0.97145812715112 | 0.867446819099186 |
| | 0.446026618800274 | 0.309851147790034 |
| | 1.68780498277243 | 0.445321205572138 |
| | 0.232277886568413 | 0.707688665292451 |
| | 2.928158640916779 | 0.753596263903473 |
| | 0.663417152211545 | 0.198079326258973 |
| | 0.315332268819816 | 0.384002662116935 |
| | 2.062088452154218 | 1.435688454955132 |
| | 1.310833744681102 | 1.324232751860854 |
| | None | None |
| | None | None |
| | 0.225412467462684 | 0.456276962128364 |
| | 0.269951982631293 | 0.239974866141399 |
| | 0.174503843494418 | 0.797050322264493 |
| | 0.409388141286222 | 0.894500824786353 |
| | 0.571483288988989 | 0.871487403971925 |
| | 0.215255979257102 | 0.46738820039279 |
| | 0.0862967906730772 | 0.496976389189007 |
| | 0.257863810145652 | 0.554067717885896 |
| | 0.452835735404153 | 2.014464270864558 |
| | 0.498176791514992 | 1.496596408046041 |
| | None | None |
| | None | None |
| | 0.319734475687953 | 0.882989518515763 |
| | 0.33391937667266 | 0.205463882600825 |
| | 0.944227049347657 | 0.461850462405648 |
| | 0.334262569630727 | 0.998453550194608 |HBV-HCC
HCV-HCC
HDV-HCC
HBV-HCC
HCV-HCC
HDV-HCC
c
hPVT1
### Chart
| Category | non-tumor | tumor |
|---|---|---|
| | 2.820024284886351 | 4.655699962810284 |
| | 0.25748835394185 | 2.440072622549938 |
| | 1.314826178824706 | 4.33043840091949 |
| | 7.423927319865816 | 7.052496704039648 |
| | 0.713353996818545 | 2.135512120918162 |
| | 3.367146720152593 | 6.55944879269448 |
| | 3.962895945177717 | 9.86079583307385 |
| | 1.36258486446522 | 2.776894922225159 |
| | 1.824581490173245 | 2.375832432747308 |
| | 0.16665841695149 | 0.435981277287457 |
| | 0.35587489972521 | 1.848479554747714 |
| | None | None |
| | None | None |
| | 3.615734100697887 | 0.818952162137302 |
| | 0.761937510499531 | 0.726823558056161 |
| | 0.674306816341331 | 0.729698344906812 |
| | 5.143154834298487 | 6.451229256257881 |
| | 0.71316464403755 | 0.274560708623859 |
| | 5.548385720949465 | 1.013058921507771 |
| | 9.193983560864798 | 9.091217958979035 |
| | 5.775715126965077 | 4.032297507850141 |
| | 3.505308903485347 | 4.763076059215534 |
| | 1.849066468827463 | 4.059068217826362 |
| | None | None |
| | None | None |
| | 1.901104170930877 | 1.79838148363061 |
| | 0.848258764516074 | 0.241236997032576 |
| | 4.541889435606457 | 4.488936183807852 |
| | 4.848199569406979 | 4.370069083696973 |HBV-HCC
HCV-HCC
HDV-HCC
Figure S2. The lncRNAs, DBH-AS1, hDREH and hPVT1 were differentially dysregulated in HBV-, HCV-, and HDV-related HCC. The expression of lncRNAs in the tumor and surrounding non-tumorous tissue in a series of patients with HCC-associated with HBV, HCV and HDV was analyzed by qRT-PCR. The expression levels of lncRNAs was normalized to the house keeping gene GAPDH. Y axis indicate the relative expression of lncRNAs, which was calculated as described in the Materials and Methods. (a) DBH-AS1; (b) hDREH; (c) hPVT1.
